# Repurposing Mercaptopurine Through Collateral Lethality to Treat Cancers with Somatic *RB1–NUDT15* Loss

**DOI:** 10.1002/mco2.70361

**Published:** 2025-09-01

**Authors:** Tao Zhou, Huayun Yan, Dandan Yin, Yun Deng, Huancheng Fu, Zichen Zhao, Shuang Li, Xiaoxi Lu, Yiqi Deng, Hai‐Ning Chen, Wei‐Han Zhang, Yunying Shi, Yangjuan Bai, Bei Cai, Lanlan Wang, Zhaoqian Liu, Wei Zhang, Lili Jiang, Yang Shu, Bo Liu, Yan Zhang, Heng Xu

**Affiliations:** ^1^ Department of Laboratory Medicine/Research Centre of Clinical Laboratory Medicine West China Hospital Sichuan University Chengdu Sichuan China; ^2^ State Key Laboratory of Biotherapy and Cancer Center West China Hospital Sichuan University Chengdu China; ^3^ Department of Medical Oncology Lung Cancer Center/Lung Cancer Institute West China Hospital Sichuan University Chengdu Sichuan China; ^4^ Department of Pathology West China Hospital Sichuan University Chengdu Sichuan China; ^5^ Department of Pediatric Hematology/Oncology West China Second Hospital Sichuan University Chengdu Sichuan China; ^6^ Department of General Surgery Colorectal Cancer Center West China Hospital Sichuan University Chengdu Sichuan China; ^7^ Institute of General Surgery West China Hospital Sichuan University Chengdu Sichuan China; ^8^ Department of General Surgery Gastric Cancer Center West China Hospital Sichuan University Chengdu Sichuan China; ^9^ Department of Nephrology West China Hospital Sichuan University Chengdu Sichuan China; ^10^ Department of Clinical Pharmacology Hunan Key Laboratory of Pharmacogenetics Xiangya Hospital Central South University Changsha Hunan China; ^11^ Tianfu Jincheng Laboratory Chengdu Sichuan China

**Keywords:** collateral lethality, copy number deletion, drug repurposing, mercaptopurine, NUDT15, RB1

## Abstract

Somatic retinoblastoma 1 (*RB1*) loss is prevalent across different cancer types and is enriched in treatment‐refractory tumors, such as castration‐resistant prostate cancer (CRPC) and small‐cell lung cancer, but cannot be considered as a direct druggable target. In this study, we revealed that the close proximity of nudix hydrolase 15 (*NUDT15*) and *RB1* may result in their common somatic codeletion or epigenomic cosilencing in different cancer types and subsequent significant positive correlations of their expressions at the bulk transcriptional and single‐cell levels. With clinical CRPC samples, co‐loss of *RB1* and *NUDT15* were commonly observed (14 out of 21). Due to the contribution of *NUDT15* deficiency to thiopurine‐induced toxicity, exploiting a vulnerability conferred by *RB1–NUDT15* loss raised the possibility of repurposing thiopurine (e.g., mercaptopurine) for precise therapeutics. A positive relationship between *RB1*/*NUDT15* ploidy score and mercaptopurine drug sensitivity was found in 543 cancer cell lines. Experimentally, knocking‐down *NUDT15* sensitizes the cancer cell lines to mercaptopurine treatment by inhibiting cell cycle progression and increasing apoptosis, but does not induce mercaptopurine‐related leucopenia in xenograft model. Our study elucidates the molecular basis for precise mercaptopurine therapy in RB1‐deficient tumors and demonstrates how leveraging collateral lethality alongside drug repurposing uncovers targetable vulnerabilities in stratified patient cohorts.

## Graphical abstract

In this study, we found the close proximity of *NUDT15* and *RB1* in terms of chromosome location and exploited the fact that NUDT15 deficiency induces 6MP‐related adverse drug reactions, thus providing a possible collateral lethality (a specific type of synthetic lethality) strategy to treat RB1‐deficient tumors (created with www.biorender.com).

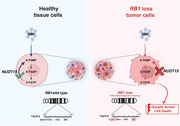

## 1 Introduction

Functional inactivation induced by somatic mutations (mostly nonsense and frameshift) and copy number (CN) deletion of the retinoblastoma tumor suppressor gene (TSG) (*RB1*), which encodes the retinoblastoma protein (RB), is commonly identified among diverse cancer types [1, 2, 3], resulting in dysfunction of cell cycle progression via deregulation of the activity of E2F transcription factors and promotion of subsequent tumor‐related gene expression [4]. Among all cancer types, *RB1* alterations are more commonly identified in treatment‐refractory tumors with neuroendocrine or small‐cell features (e.g., small‐cell lung cancer), which may arise in multiple organs and indicate a poor prognosis [3, 5]. With a landscape of investigation on *RB1* in 22,432 Chinese patients with solid cancers, ∼8% of whom carry somatic *RB1* alterations, including mutations and deletions of CN [3]. In prostate cancer (PRCA), CN deletions are more prevalent than they are in other types of cancer [6]. Accumulating genomic evidence revealed a homozygous *RB1* deletion in ∼5–10% of primary PRCA, which increased to ∼60% in metastatic and androgen receptor inhibitor‐resistant/castration‐resistant PRCA (CRPC), along with increased neuroendocrine features [7, 8]. Therefore, the prevalent CN loss of *RB1* in PRCA is associated with poor prognosis and exhibited as the only potential prognostic factor at the genomic level [9, 10, 11]. Given the critical need for effective strategies to treat metastatic CRPC, clinical trials and novel therapies have been conducted [12, 13, 14]. However, investigation is lacking on the association of *RB1* deletion with the therapeutic efficacy of these strategies, which are specific for PRCA rather than other tumors with *RB1* alterations.

On the other hand, since RB1 cannot be considered as a therapeutic target, genes involved in RB1‐deficiency‐related aberrant signals and pathways were considered as actionable targets, such as transcriptional targets and tumor microenvironment [15, 16, 17]. At genomic level, the majority of somatic CN deletion induces simultaneous deletion of the passenger genes chromosomally adjacent to the driver genes (e.g., *RB1*), thereby facilitating “collateral lethality” as a new therapeutic strategy to target the neighboring genes for cancer treatment [18, 19, 20]. Collateral lethality arises when the chromosomal codeletion of functionally redundant passenger genes adjacent to tumor suppressor loci creates cancer‐specific vulnerabilities. Unlike classical synthetic lethality that targets unrelated genetic interactions, collateral lethality exploits cancer cells' intrinsic genomic instability to design precision therapies [20]. Previous studies have demonstrated collateral lethality effectively targeting cancers harboring deletions adjacent to tumor suppressors such as SMAD4/ME2 [19], metabolic paralog pairs ENO1/ENO2 [21] and MTAP/PRMT5 [22], highlighting their potential in clinical translation. However, the involved drugs are not US Food and Drug Administration (US FDA)‐approved for clinical use, thus requiring combining genomic mapping of passenger gene deletions with drug repurposing strategies to identify clinically actionable collateral lethal pairs.

In fact, codeletion of a *RB1* neighbor gene (i.e., *SUCLA2*) was considered as a pharmacologically targetable vulnerability in PRCA [23]. However, the chemicals screened out from the library of natural compounds by this study have no clinical applications. Interestingly, nudix hydrolase 15 (*NUDT15*), which encodes a purine‐specific nucleotide diphosphatase, is also in close proximity to *RB1* [24]. As a substrate of NUDT15, active metabolites of thiopurine (e.g., mercaptopurine [6MP]) are essential for the treatment of pediatric patients with acute lymphoblastic leukemia. Mechanistically, it is well established that NUDT15 can dephosphorylate thiopurine active metabolites to prevent cytotoxicity induced by their incorporation into DNA and RNA [24]. Due to the narrow therapeutic index of thiopurine, however, dose‐dependent adverse drug reactions (e.g., leukopenia) have long been observed in a small proportion of patients taking standard medication. Using pharmacogenetic approaches, a series of germline variants in *NUDT15* that cause thiopurine intolerance through disrupting the enzymatic activity or downregulating the expression level of *NUDT15* have been identified, resulting in the accumulation of the toxic thiopurine metabolites in both normal and leukemia cells [24, 25, 26]. Therefore, germline variants in *NUDT15* have been labeled as pharmacogenetic biomarkers for thiopurine use, which is a prime example of precision medicine [27]. Moreover, according to the preclinical evaluation of NUDT15‐guided thiopurine therapy, somatic knockout of *NUDT15* can significantly increase the antileukemic efficacy of 6MP [26, 28]. Given the possible codeletion of *RB1* and *NUDT15* in a variety of cancer types, a thiopurine‐based collateral lethality strategy could be practical to precisely treat cancer patients with somatic deletion at the *RB1–NUDT15* locus, which is also a potential drug repurposing strategy for thiopurine in cancer treatment.

In this study, we aim to determine the prevalence of somatic deletions of *RB1* and *NUDT15* across various cancer types, and to evaluate the metabolic vulnerability caused by *NUDT15* deletion in cell lines and mouse models, which will shed light on a possible 6MP‐based collateral lethality and drug repurposing strategy for treating cancers with somatic *RB1–NUDT15* deletion.

## 2 Results

### 2.1 Somatic *RB1–NUDT15* Deletion Across Cancer Types

**FIGURE 1:**
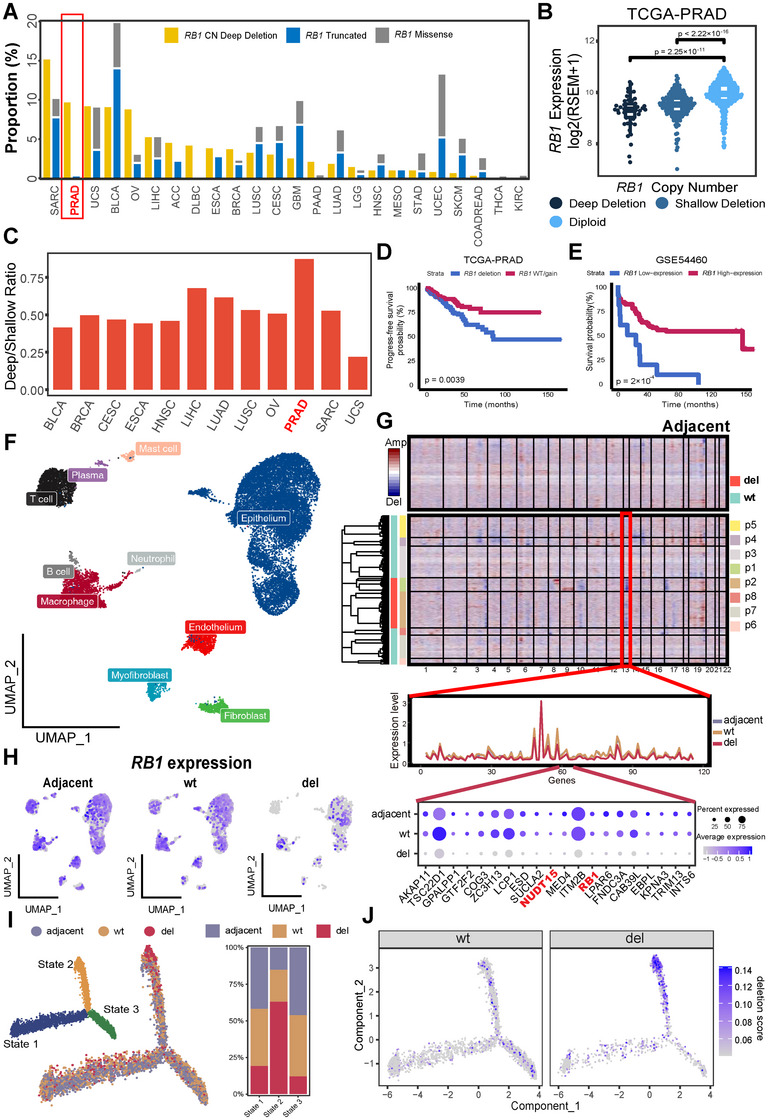
Frequencies and impact of *RB1* alterations among different cancers. (A) The frequency of *RB1* CN deletion and mutations among different cancer types in TCGA. (B) Impact of *RB1* CN deletions on *RB1* expression in TCGA–PRCA. (C) Comparison of *RB1* expression between *RB1* deep and shallow deletion among different TCGA cancer types. (D) Prognostic association of *RB1* CN deletion in TCGA–PRCA. (E) Prognostic association of *RB1* expression in cohort without CN deletion information (i.e., GSE54460). (F) Distribution of different cell clusters in prostate cancer visualized by UMAP. (G) CN estimated by InferCNV and the illustration of *RB1* deleted region. (H) *RB1* expression in each cluster separated by *RB1* deletion status. (I) Left: activation trajectory of epithelial cells and three states division, Right: components of the adjacent and *RB1* wt/deletion tumor in each state. (J) Distribution of *RB1* deletion score along the trajectory. CN, copy number; UMAP, Uniform Manifold Approximation and Projection; TCGA, The Cancer Genome Atlas; PRCA, prostate cancer; BLCA, bladder cancer; KIRC, kidney cancer; UCEC, uterine corpus endometrial carcinoma; SARC, sarcoma; UCS, uterine carcinosarcoma; OV, ovarian adenocarcinoma; LIHC, liver hepatocellular carcinoma; ACC, adrenocortical carcinoma; DLBC, lymphoid neoplasm diffuse large B‐cell lymphoma; ESCA, esophageal carcinoma; BRCA, breast invasive carcinoma; LUSC, lung squamous cell carcinoma; CESC, cervical squamous cell carcinoma and endocervical adenocarcinoma; GBM, glioblastoma multiforme; PAAD, pancreatic adenocarcinoma; LUAD, lung adenocarcinoma; LGG, brain lower grade glioma; HNSC, head and neck squamous cell carcinoma; STAD, stomach adenocarcinoma; SKCM, skin cutaneous melanoma; COADREAD, colon and rectum adenocarcinoma; THCA, thyroid carcinoma.

Given the important role of *RB1* deficiency in cancer development and progression, numerous genomic studies, such as The Cancer Genome Atlas (TCGA), have revealed the prevalence of somatic alterations (e.g., mutations and CN loss) in the *RB1* gene. Approximately 10% of somatic CN deep deletions (referring to homozygous loss) were identified in five types of cancer, including PRCA and bladder cancer (BLCA) (Figure 1A). In particular, deletion of the *RB1*‐containing region predominates over *RB1* truncating mutations (Figure 1A), and is the most prevalent deletion in PRCA according to the TCGA database and independent PRCA cohorts and other cancer types in Chinese patients (Figure S1A–C). Not surprisingly, both shallow (referring to heterozygous) and deep deletions result in enrichment of the RB pathway and decreased expression of *RB1* (Figures 1D and S1D), which can be validated in other cancer types from TCGA (Figure S2). Intriguingly, PRCA has the highest average ratio of shallow to deep deletion expression (Figure 1C), indicating that the intact allele may be frequently silenced epigenetically. Moreover, both *RB1* deletions and low expression are associated with poor prognosis of PRCA in TCGA and other independent patient cohorts (Figures 1D,E and S3A,B), which is consistent with the increased prevalence of *RB1* deletions in metastatic PRCA and CRPC. Besides, *RB1* deletions have a negative effect on the treatment outcomes of multiple other cancer types, including kidney cancer and uterine corpus endometrial carcinoma (UCEC) (Figure S3C).

### 2.2 Distinct Single‐Cell Profile of PRCA with *RB1* Deletion

To investigate the distinct profile of *RB1* deleted cancer cells, we performed single‐cell transcriptome analysis using a publicly available resource [29]. After quality control, a total of 20,318 cells were acquired from 15 samples (seven adjacent and eight tumor samples from 11 patients, including four paired adjacent and tumor samples) (Figure S4A). With unsupervised clustering, cells were divided into 10 major clusters (Figure 1F) and annotated based on canonical markers (Figure S4B), including epithelial and nine clusters of the tumor microenvironment. CN estimation of epithelial cells revealed that two out of eight tumor samples contained a broad deletion in chromosome 13, which led to the loss of *RB1* and its neighboring genes (Figure 1G). Consequently, the frequency of *RB1* ^+^ cells was lower in *RB1*‐deleted epithelial cells than in adjacent and *RB1*‐CN‐wildtype tumors (Figure 1H). Using evolutional trajectory analysis, two distinct activation paths from normal epithelial to malignant epithelial cells were identified. Three states of epithelial cells were defined, and *RB1*‐deleted samples were enriched in State 2 (Figure 1I). For individual cells, the high deletion score of chr13 gradually increased along the activation trajectory from State 1 to State 2 rather than to State 3 (Figure 1J). Consistently, *RB1* ^+^ cells tend to evenly distribute across three states (Figure S4C), whereas the proportion of *RB1* ^+^ cells is lower in *RB1*‐CN‐wildtype samples compared with *RB1*‐deleted samples in each state, particularly in State 2 (Figure S4D). Moreover, mutual interactions between different tumor microenvironment clusters and epithelial cells are more prolific in adjacent and *RB1*‐CN‐wildtype samples than in *RB1*‐deleted samples (Figure S4E). A series of ligand‐receptor pairs are abundant in both adjacent and *RB1*‐CN‐wildtype or *RB1*‐CN‐wildtype alone, with the exception of the SPP1–CD44 interaction (Figure S4F), which is involved in tissue damage in the immune response to cancer and poor prognosis in cancer [30, 31]. Using our integrated single‐cell transcriptome data [32], we also identified common *RB1* deletions and distinct evolutionary enrichment at the pan‐cancer level (Figure S5A–C).

### 2.3 Co‐Occurrence of *NUDT15* Deletion at *RB1–NUDT15* Locus

**FIGURE 2:**
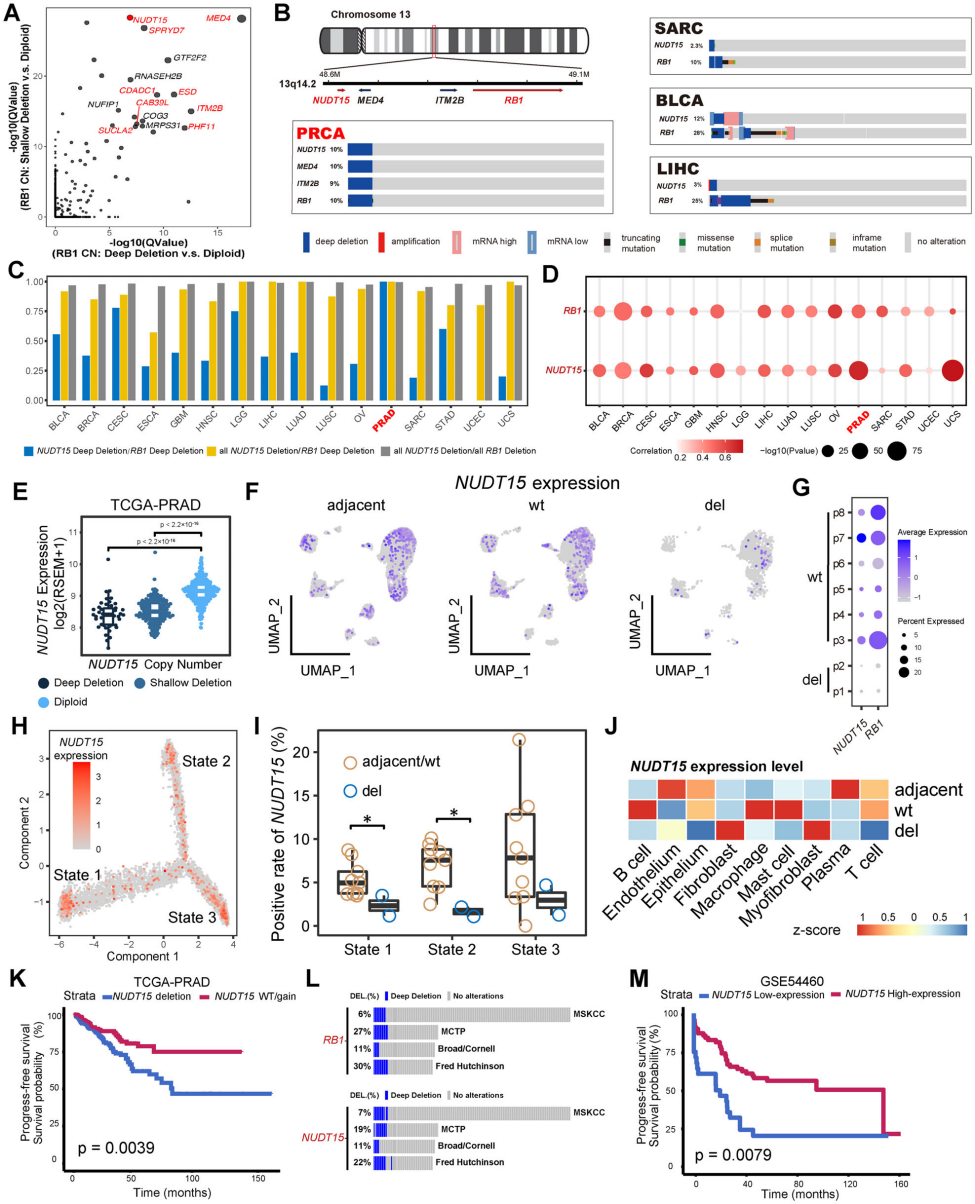
Chromosomal proximity and codeletion of *NUDT15* and *RB1*. (A) Overlapped differential expressed genes of CN deep/shallow deletion versus diploid TCGA–PRCA tumor. (B) chromosomal proximity and common cooccurrence of deep deletion of *NUDT15* and *RB1* in TCGA–PRCA, SARC, BLCA, and LIHC. (C) Ratio of *NUDT15* deletion versus *RB1* deletion among different cancer types. (D) Correlation of deletion status with expression for *NUDT15* and *RB1* respectively across different TCGA cancer types. (E) *NUDT15* expression in each cluster separated by *NUDT15* deletion status. (F) *NUDT15* expression in each cluster separated by chr13 deletion status in prostate cancer single cell cohort. (G) Illustration of *NUDT15*–*RB1* codeletion in each patients from the single cell cohort. (H) *NUDT15* expression along the trajectory. (I) Comparison of *NUDT15* ^+^ epithelial cells rate in each state separated by adjacent/wt versus chr13 deleted samples. (J) *NUDT15* expression of different cell components in in different sample origins. CN, copy number; chr13, chromosome 13; (K) Prognostic association of *NUDT15* deletion in TCGA–PRCA; (L) Frequencies and codeletions of *RB1* and *NUDT15* in independent prostate cancer cohorts; (M) Prognostic association of *NUDT15* expression in independent prostate cancer cohort without CN deletion information (i.e., GSE54460).

Next, we investigated the differentially expressed genes (DEGs) induced by *RB1*‐deletion in PRCA. Interestingly, a series of top ranked DEGs were in close chromosomal proximity to *RB1*, including *NUDT15* (Figure 2A,B). Although multiple genes can undergo codeletion with RB1 at this locus, only NUDT15 encodes a protein directly linked to the metabolism of a clinically approved drug. Specifically, the enzymatic deficiency of NUDT15 is well established to significantly enhance the cytotoxicity of 6MP, an US FDA‐approved therapeutic agent [24, 33]. This characteristic positions NUDT15 as an ideal candidate gene for exploring a collateral lethality‐based therapeutic strategy through drug repurposing, facilitating rapid clinical translation. Indeed, co‐occurrence of *NUDT15* and *RB1* deep deletion is commonly observed in multiple cancer types according to the TCGA database (Figure 2B), and nearly all patients with deep/shallow *RB1* deletion also carry *NUDT15* deletion across cancer types, which is induced by the extensive deletion event at this locus (Figure 2C). Not surprisingly, expression of both *RB1* and *NUDT15* is correlated with deletion status (Figures 2D,E and S6), and no significant difference in *NUDT15* expression was observed between deep and shallow deletion in PRCA (Figure 2E). At the single cell level, *NUDT15* was also downregulated in epithelial cells of *RB1*‐deleted samples, alongside *RB1* expression (Figure 2F,G). Similarly, lower proportions of *NUDT15* ^+^ were observed in *RB1*‐deleted samples compared with adjacent and *RB1*‐CN‐wildtype samples, and this difference was statistically significant in State 1 and 2 (Figures 2H,I). In contrast, downregulation of *NUDT15* was not specifically detected in *RB1*‐deleted samples because the CN was intact for tumor microenvironment cells (Figure 2J).

*NUDT15* deletion can predict poor progress‐free survival in PRCA due to its perfect co‐occurrence with *RB1* deletion (Figure 2K), which is also present in other independent PRCA cohorts (Figure 2L). Consistently, a significant association between *NUDT15* deletion and poor survival was also observed in other cancer types (e.g., UCEC), with a similar pattern of *RB1* deletion (Figure S7A–C). Considering the impact of CN deletion on gene expression, *NUDT15* expression is associated with poor prognosis in PRCA patient cohorts with transcriptional information (e.g., GSE54460) (Figures 2M and S7D).

### 2.4 CN‐Independent Correlation of *NUDT15* and *RB1* Expression

**FIGURE 3:**
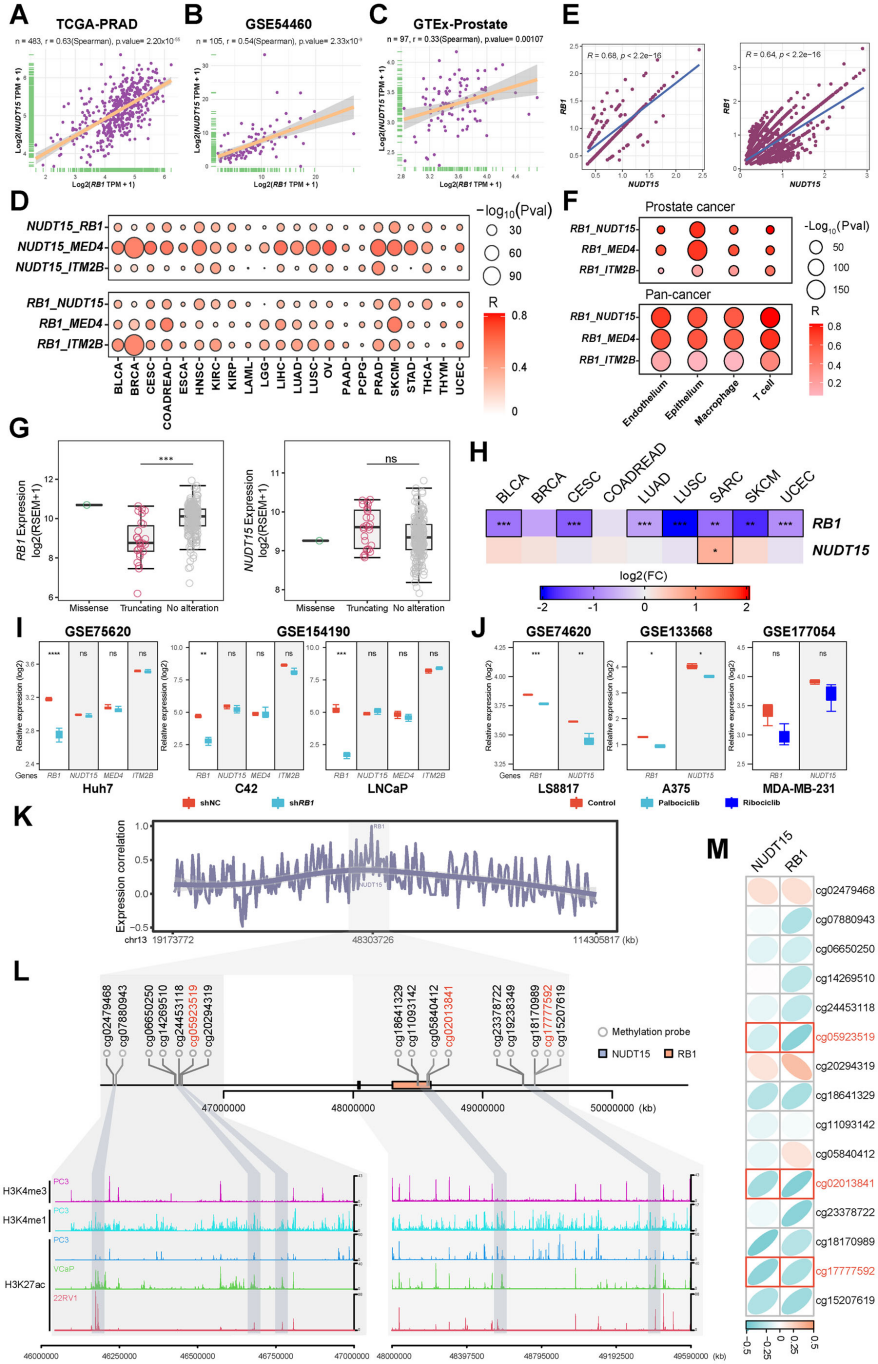
Deletion‐independent expression correlation of *NUDT15* and *RB1*. (A–C) Correlation of *NUDT15* and *RB1* expression in tumor samples from TCGA and an independent prostate cancer cohort, as well as normal prostate tissues from GTEx. (D) Correlation of *NUDT15* and *RB1* expression with their neighbor genes across different TCGA cancer types. (E and F) Correlation of *NUDT15* and *RB1* expression in epithelial cells and tumor microenvironment cell components at single cell resolutions in prostate cancer and pan‐cancer patients. (G and H) Impact of *RB1* mutation status *NUDT15*/*RB1* expression across TCGA cancer types. (I) Impact of *RB1* downregulation on the expression of its neighbor genes in cell lines. (J) Impact of CDK4/6 inhibitor induced *RB1* downregulation on *NUDT15* expression. (K) Correlation of gene expression between RB1 and other genes located on chromosome 13, highlighting potential enhancer regions. (L) Identification of candidate enhancer regions based on enrichment of H3K27ac and H3K4me1 signals (markers of active enhancers) and absence of H3K4me3 (a promoter‐specific marker). (M) Correlation analysis between DNA methylation levels at the identified enhancer regions and the expression of RB1 and NUDT15. *, *p* < 0.01; ***, *p* < 0.0001; ns, not significant.

Interestingly, a strong correlation between *NUDT15* and *RB1* expression was observed in PRCA (Figure 3A), which can be validated in multiple PRCA cohorts (Figures 3B and S8A–F). However, this correlation cannot be fully explained by somatic deletion of the *RB1–NUDT15* locus, as it is also present in adjacent/normal prostate samples and is independent of deletion status (Figures 3C and S8G), as well as in tumors from all cancer types after excluding *RB1*‐deleted samples (Figure 3D). At single cell level, *RB1* expression is significantly correlated with *NUDT15* expression in *RB1* ^+^ *NUDT15* ^+^ epithelial and other tumor microenvironment cells from PRCA and pan‐cancer patients (Figure 3E,F). To determine whether this correlation is induced by the regulatory role of *RB1*, we analyzed the effect of somatic *RB1* mutations on *NUDT15* expression. Mechanistically, a truncating mutation can induce not only RB1 loss‐of‐function, but also decreased *RB1* expression via nonsense‐mediated decay. In samples with *RB1* truncating mutations in BLCA and other cancers with frequent *RB1* truncating mutations (few have been identified in PRCA), *RB1* but not *NUDT15* expression was significantly decreased after excluding *RB1*‐CN‐deleted samples (Figure 3G,H). According to the public resource, the expression of *NUDT15* was not affected when *RB1* was posttranscriptionally downregulated in multiple cell lines, indicating no direct regulatory role of RB1 on *NUDT15* expression (Figure 3I). In contrast, regulation of *RB1* at the transcriptional level may simultaneously affect *NUDT15* expression. For instance, treatment with CDK4/6 inhibitors (e.g., palbociclib) may induce dephosphorylation and/or downregulation of *RB1* in specific cancer types [34, 35], which is accompanied by decreased *NUDT15* expression in various cell lines according to the public databases (e.g., GSE74620, GSE133568, and GSE177054) (Figure 3J). Furthermore, expression of the other two neighbors of *RB1* (i.e., *MED4* and *ITEM2B*) was also correlated to both *RB1* and *NUDT15* expression, but not regulated by *RB1* either (Figure 3D,I). It is assumed that the strong correlation between neighboring genes may be caused by the shared genomic regulatory elements within the same cluster, which can also be observed in other loci, such as *ACTB* (neighbor with *FSCN1* and *RNF216*) and *GAPDH* (neighbor with *NCAPD2* and *MRPL51*) (Figure S8H–J). To investigate potential epigenetic mechanisms underlying the coordinated expression of RB1 and NUDT15, we first examined expression correlations across chromosome 13 and identified a cluster of genes exhibiting strong coexpression with RB1, including NUDT15 (Figure 3K). This pattern suggested the presence of shared regulatory elements in the local genomic region. By integrating 450K DNA methylation microarray data from TCGA–PRAD cohorts and publicly available ChIP‐seq datasets from three PRCA cell lines (22RV1, PC‐3, and VCaP), we further explored this possibility. Several candidate enhancer regions proximal to the RB1 and NUDT15 loci were identified, characterized by enrichment of H3K27ac and H3K4me1 signals (activation signal for enhancer or promoter) and a lack of H3K4me3 signal (specific signal for promoter) (Figure 3L). Notably, the methylation levels of several CpG sites within these regions (e.g., cg05923519, cg02013841 and cg17777592) showed consistent and significant negative correlations with the expression of both RB1 and NUDT15 (Figures 3M and S8K–M). These findings suggested shared enhancer elements may mediate epigenetic coregulation of the two genes. In fact, such cluster‐by‐cluster basis has been reported previously, which may be induced by topologically associated domains [36, 37]. Nevertheless, CN‐independent *RB1* somatic silencing results in a higher proportion of *NUDT15* downregulation in tumors.

### 2.5 Co‐loss of *NUDT15* and *RB1* in CRPC

**FIGURE 4:**
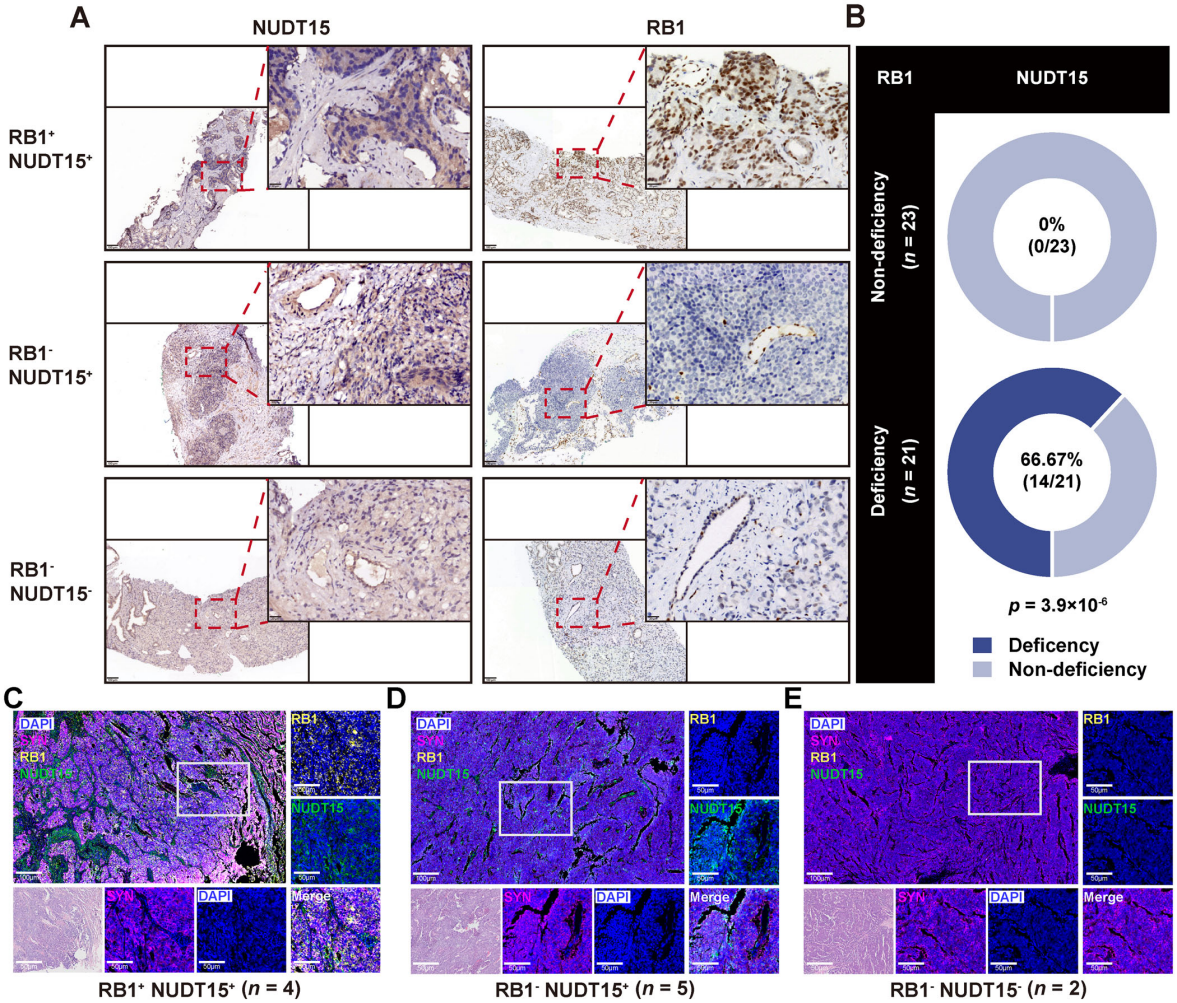
IHC validation of RB1/NUDT15 co‐loss in CRPC patients. (A) Representative IHC staining of CRPC patients with different RB1 and NUDT15 status. (B) Statistical result of the correlation of NUDT15 and RB1 in 44 CRPC patients. IHC, immunohistochemistry; CRPC, castration resistant prostate cancer. (C–E) Representative multiplex immunofluorescence staining of SCLC patients with different RB1 and NUDT15 status.

To estimate the frequency of co‐loss of NUDT15 and RB1 in clinical samples, we performed immunohistochemistry (IHC) in 44 CRPC samples, including 21 have been determined as RB1 loss and 23 with intact RB1 expression as control. No NUDT15 loss were detected in the tumor cells of 23 RB1 expressed samples, whereas 14 out of 21 (66.7%) RB1‐loss samples exhibited co‐loss of NUDT15 in tumor cells with *p* = 3.9 × 10^−6^ (Figure 4A,B), supporting the frequent co‐loss of NUDT15 in CRPC patients with RB1 loss. To further validate the prevalence of *RB1*–*NUDT15* codeletion across tumor types, we analyzed archived small cell lung cancer (SCLC) specimens via IHC. Among 10 available SCLC samples, concurrent loss of RB1 and NUDT15 protein expression was observed in two cases (20%), suggesting that codeletion also occurs in a subset of SCLC patients. This finding extends the applicability of the proposed collateral lethality strategy beyond PRCA and supports its potential relevance in other RB1‐deficient malignancies (Figure 4C–E).

### 2.6 Association of 6MP Sensitivity with *RB1–NUDT15* Deletion in Cancer Cell Lines

**FIGURE 5:**
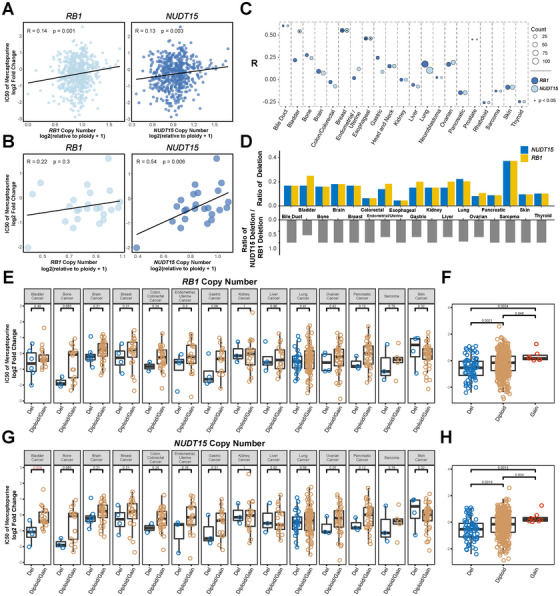
Impact of *RB1*–*NUDT15* deletions on the sensitivity of 6MP in cancer cell lines. (A–C) Correlation of ploidy score of *RB1/NUDT15* with IC50 of 6MP in all cancer cell lines, bladder cancer cell lines, and each cancer type cell lines separately. (D) Ploidy score based *RB1*/*NUDT15* deletion frequencies in each cancer type cell lines. (E and F) Association of *RB1* deletion with 6MP sensitivity in each cancer type and overall cell lines. (G and H) Association of *NUDT15* deletion with 6MP sensitivity in each cancer type and overall cell lines. 6MP, mercaptopurine.

Given the well‐established causal relationship between the NUDT15 enzyme activity and metabolism of thiopurine (e.g., 6MP) and the increased antileukemic efficacy of 6MP for *NUDT15* deficient leukemia at cell line and mouse levels [24, 26], we hypothesized that somatic deletion of *NUDT15* could also sensitize the solid cancer cells to 6MP. With genomic and drug sensitive information retrieved from the public databases (Depmap: https://depmap.org/portal/, and cBioPortal: https://www.cbioportal.org/), we observed significant positive correlations between 6MP IC_50_ and *RB1*/*NUDT15* ploidy score in 543 cell lines from 21 cancer types (Figure 5A). To exclude the influence of cancer type, we conducted separate correlation analyses for each cancer type. Interestingly, due to the imperfect match of *NUDT15* and *RB1* deletion in BLCA cell lines, the IC_50_ of 6MP is only related to the ploidy score of *NUDT15* but not *RB1*, confirming the causal relationship impact of *NUDT15* but not *RB1* on 6MP sensitivity (Figure 5B). The statistically significant association between IC_50_‐based 6MP sensitivity and NUDT15 CN was only reached in a few cancer types, including BLCA, breast cancer and esophageal cancer. Nonetheless, modest correlations were noticed in terms of R score, including PRCA and bile duct cancer (Figure 5C). To further confirm the specificity of 6MP for *RB1*–*NUDT15* codeleted tumors, we systematically screened all available drugs in the DepMap database to identify potential alternative candidates. Although several compounds exhibited comparable or slightly stronger correlations with *RB1*–*NUDT15* deletion status than 6MP, most of these agents lack clinical approval or are not routinely utilized for cancer therapy (Figure S9A–D). For example, orbifloxacin is approved solely for veterinary use, while flucytosine is clinically approved exclusively for treating infectious diseases. Given the uncertainties regarding the therapeutic dosages, safety profiles, and clinical efficacies of repurposing these agents for cancer treatment, 6MP remains the most viable candidate for clinical translation in treating *RB1*–*NUDT15* codeleted cancers due to its established safety profile and comprehensive clinical guidelines. After categorizing cell lines into deletion and CN‐intact based on ploidy score, CN loss is commonly identified in these cell lines, and *RB1*‐deletion and *NUDT15*‐deletion are largely matched, consistent with the observation in patient tumor samples (Figure 5D). Moreover, 6MP IC_50_ tends to be lower in *RB1–NUDT15* deleted cells in the majority of cancer types and reached statistical significance in the combined cell lines (Figure 5E–H), indicating that *RB1*‐deleted tumors may be treatable via collateral lethality.

### 2.7 Impact of *NUDT15* Downregulation on 6MP Sensitivity in Cancer Cell Lines

**FIGURE 6:**
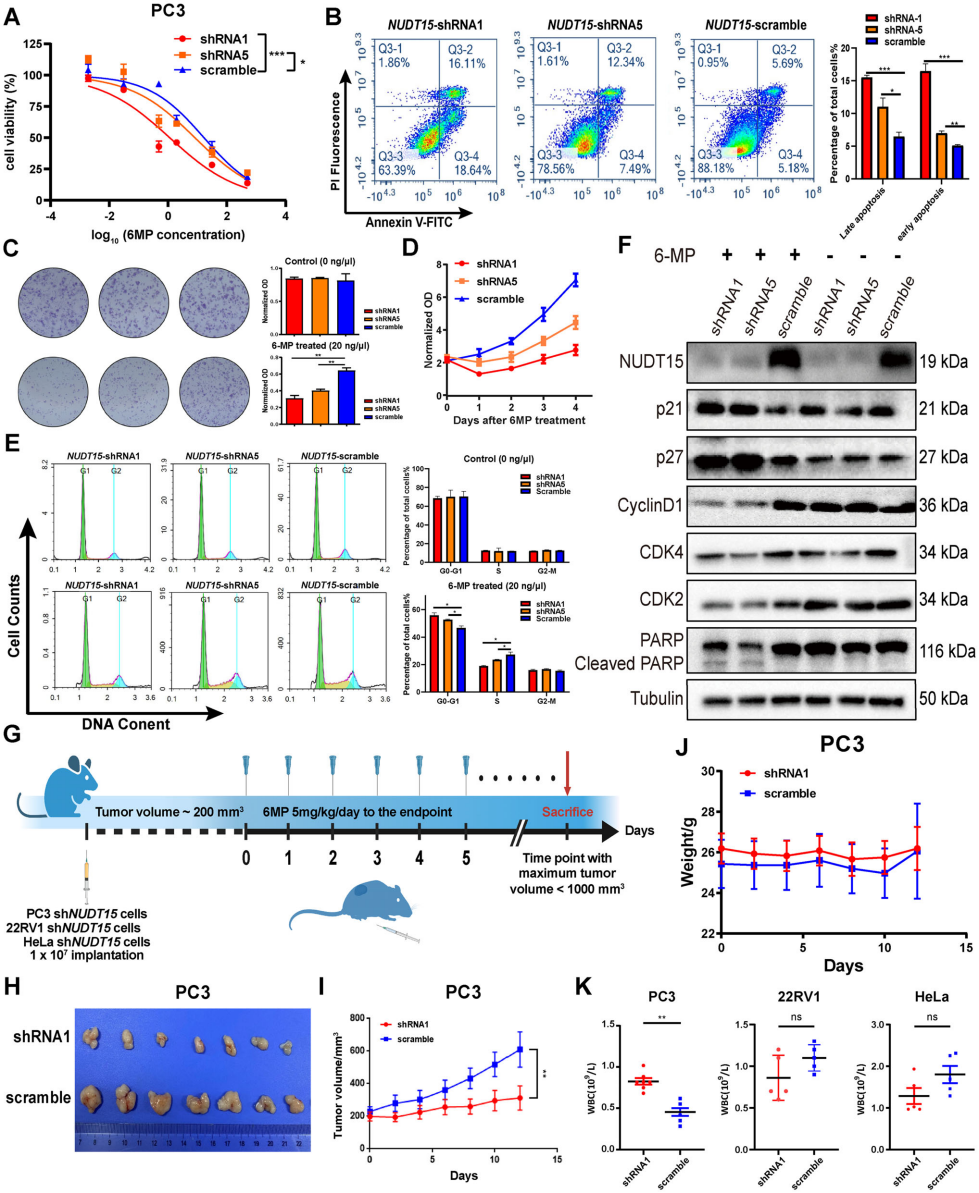
Experimental validation of *NUDT15* effect on 6MP sensitivity. (A) 6MP sensitivity evaluation on *NUDT15*‐KD/control in PC3 cell line, three replicates were performed for each concentration. (B) Apoptosis assay of *NUDT15*‐KD/control PC3 cells after 6MP treatment detected by Annexin V‐PI double staining. (C) The effect of *NUDT15*‐KD on clone formation of PC3 cells after 6MP treatment. (D) The effect of *NUDT15*‐KD on cell proliferation of PC3 cells after 6MP treatment. (E) The effect of *NUDT15*‐KD on each cell cycle of PC3 cells before/after 6MP treatment. (F) Cell cycle related proteins’ changes in 6MP‐treated *NUDT15*‐KD PC3 cells. (G) A schematics of the experimental design of xenograft. (H and I) Illustration of the final tumor and determination of subcutaneous tumor volume with *NUDT15*‐KD/control PC3 cells (*n* = 7). (J) Evaluation of body weight at the end points before sacrificing the nude mice. (K) Evaluation of WBC at the end points before sacrificing the nude mice. 6MP, mercaptopurine; WBC, white blood count; KD, knockdown. *, *p *< 0.05; **, *p *< 0.01; ***, *p *< 0.001; ****, *p *< 0.0001.

To exclude the influence of different cancer type background, we performed experimental confirmation of the impact of *NUDT15* expression on 6MP sensitivity in cell lines derived from PRCA (i.e., PC3, 22RV1) and cervical cancer (i.e., HeLa), where *NUDT15* deletion is associated with a poor prognosis (Figure 2K). We established stable cell lines using two out of five shRNAs against *NUDT15* that exhibit different extents of knockdown (KD) effect (Figures S10A–D and S11A,B). Not surprisingly, decreased *NUDT15* expression significantly sensitized both cell lines to 6MP up to 15 folds in PC3 (IC_50_ = 1.139 ng/µL, 95%CI: 0.5834–2.297 in *NUDT15*‐KD vs. IC_50_ = 16.69 ng/µL, 95%CI: 10.26–27.36 in scramble, *p *< 0.001), 3.2 folds in 22RV1 (IC_50_ = 0.198 ng/µL, 95%CI: 0.143–0.276 in *NUDT15*‐KD vs. IC_50_ = 0.64 ng/µL, 95%CI: 0.532–0.775 in scramble, *p *< 0.001), and 3.1 folds in HeLa (IC_50_ = 0.018 ng/µL, 95%CI: 0.0145–0.0221 in *NUDT15*‐KD vs. IC_50_ = 0.057 ng/µL, 95%CI: 0.0500–0.0600 in scramble, *p *< 0.001) (Figures 6A, S10E, and S11C), which is consistent with the previous reports in leukemia cells [24]. Because an increase in cell proliferation can enhance the cytotoxic effect of 6MP [38], we compared the growth rate of the modified cells, and observed no difference between *NUDT15‐*KD cells and the scramble control in terms of cell proliferation assay as well as subcutaneous tumor with mouse model (Figure S10F–H), indicating that the increase in sensitivity to 6MP is primarily dependent on the KD‐induced loss of NUDT15 activity. Treating with 6MP induced a significantly higher apoptosis rate in *NUDT15*‐KD PC3 cells, but the overall apoptosis rate is low even after treating with 50 ng/µL for 72 h (Figure 6B). On the other hand, we found that 6MP treatment can also significantly separate *NUDT15*‐KD with scramble control of PC3 cells in terms of growth rate and cell cycle (Figure 6C,D). To evaluate cell death patterns, we performed Annexin V/PI staining. The majority of 6MP‐treated NUDT15‐KD cells were Annexin V‐positive, with minimal PI single‐positive staining, indicating apoptosis as the primary mode of cell death (Figure 6E). This is consistent with previously reported mechanisms of 6MP‐induced cytotoxicity [39, 40]. Accordingly, cell cycle related proteins were altered, including p21, p27, Cyclin D1, CDK2, CDK4, and cleaved PARP, which is consistent with the decreased proliferation rate and increased apoptosis rate of 6MP‐treated *NUDT15*‐KD cells (Figure 6F). Building on the phenotypic effects observed upon NUDT15 KD and 6MP treatment, we performed RNA‐seq analysis in PC3 and 22RV1 cells under different conditions. Transcriptomic profiling revealed that gene expression changes in NUDT15‐KD cells treated with 6MP were significantly enriched in pathways related to apoptosis and cell cycle regulation (Figure S12A–D). These findings are consistent with the observed increase in apoptosis and alterations in cell cycle distribution, supporting the mechanistic link between NUDT15 loss, enhanced 6MP sensitivity, and disruption of cell cycle control. To assess the potential compensatory mechanisms for NUDT15 loss in 6MP metabolism, we examined the expression of all reported 6MP metabolic pathway genes (including transporters, activating enzymes, and inactivating enzymes), using RNA‐seq data from PC3, 22RV1, and HeLa cells following NUDT15 KD (Figure S13A). No significant changes were observed. Furthermore, evaluation of these enzymes across TCGA tumor types, CCLE cell lines, and GTEx normal tissues demonstrated broadly consistent expression profiles (Figure S13B–D), suggesting minimal variability in downstream thiopurine metabolism across tumor types. These results indicate that NUDT15 loss is unlikely to be functionally compensated by alternative enzymatic pathways, and that the proposed therapeutic vulnerability is conserved across different cancer types.

Next, we evaluated the therapeutic efficacy of 6MP in vivo using a subcutaneous tumor model in nude mice (Figure 6G). A safe 6MP dosage (i.e., 5 mg/kg/day) was continuously administered once the tumor volume reached approximately 200 mm^3^, the growth rate of *NUDT15*‐KD PC3 cells was significantly attenuated compared with scramble control (Figure 6H,I), without inducing body weight loss and leucopenia in mice (Figure 6J,K), which is the most common adverse drug reaction of 6MP. Same results were observed in *NUDT15*‐KD 22RV1 cells, 6MP treatment effectively suppressed tumor growth in NUDT15 KD groups compared with controls (Figure S11D,E). Since *RB1*–*NUDT15* loss also contributes to the poor prognosis in UCEC (Figures S3C and S7A–C), we conducted similar experiments with HeLa cells, and observed that knocking‐down *NUDT15* can sensitize the cells to 6MP in vitro and in vivo, without inducing body weight loss or leucopenia (Figures S14A–C and 6K), suggesting the ubiquitous effects of NUDT15 deficiency on 6MP sensitivity in different cancer types. A slight increase in white blood cell (WBC) counts was observed in the PC3 model, while in the 22RV1 and HeLa models, WBC counts remained comparable to controls (Figure 6K). These findings suggest that 6MP effectively suppresses tumor growth in NUDT15‐deficient models without causing systemic hematologic toxicity at therapeutic doses.

**FIGURE 7:**
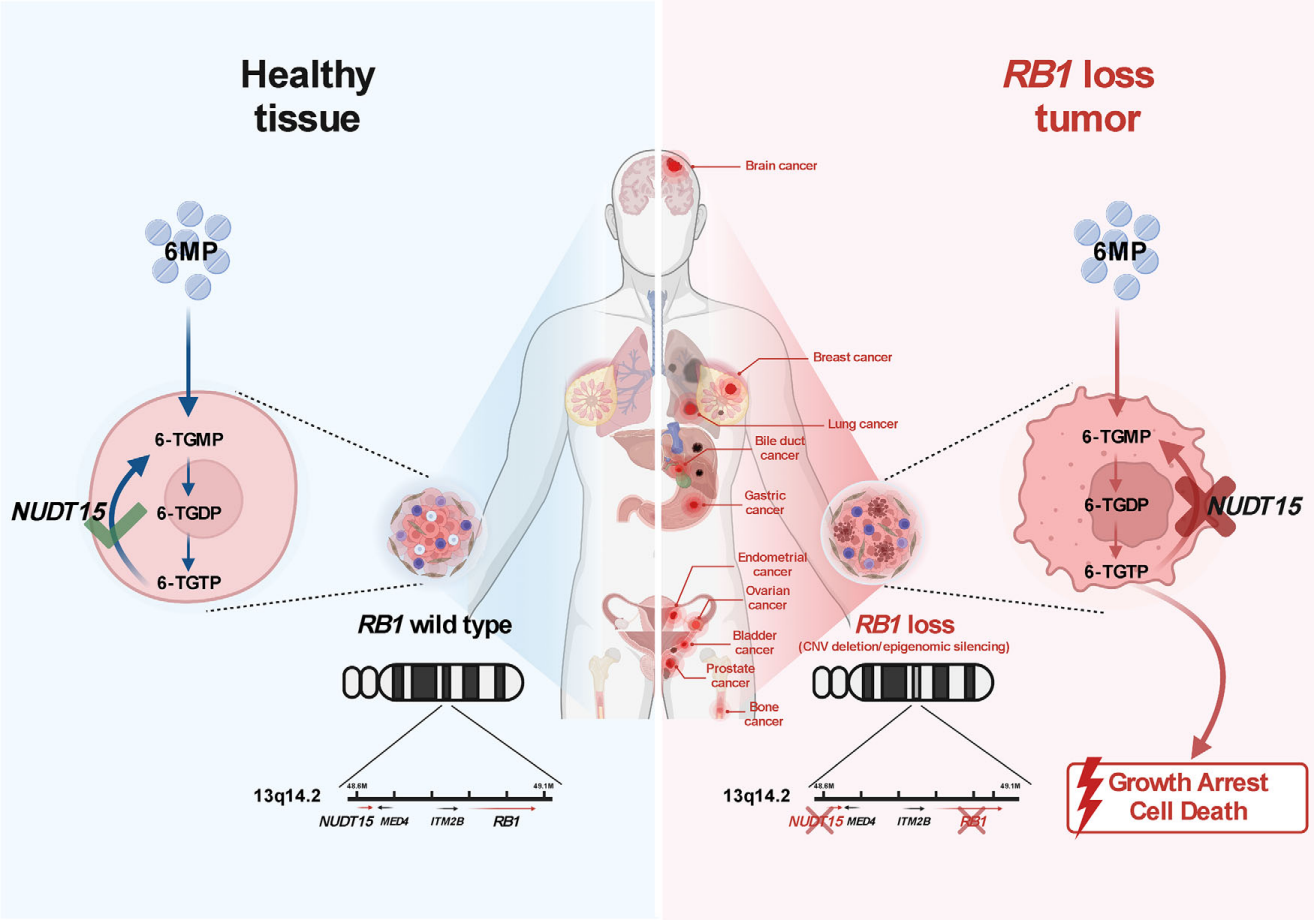
Summary of the mechanistic basis for collateral lethality and repurposing 6MPs to treat cancers with *RB1* loss (e.g., copy number deletion). NUDT15 is a critical metabolic enzyme that detoxifies the active metabolites of the US FDA‐approved drug mercaptopurine (6MP). Frequent codeletion of RB1 and NUDT15 in tumor cells impairs this detoxification mechanism due to their close chromosomal proximity, selectively sensitizing *RB1*–*NUDT15* codeleted cancer cells to 6MP‐induced cytotoxicity. Thus, it provides a collateral lethality‐based therapeutic strategy that precisely repurposes mercaptopurine to target tumors harboring *RB1*–*NUDT15* codeletion, effectively killing cancer cells while sparing normal cells. This figure was created with Biorender (www.biorender.com).

In conclusion, our findings provide a possible drug repurposing strategy with 6MP to precisely treat tumors with *NUDT15* deficiency, which is the collateral lethality partner of *RB1* (Figure 7).

## 3 Discussion

Numerous somatic alterations have been identified through large‐scale genomic characterization efforts (e.g., TCGA), aiming to provide precise cancer treatment targets. Generally, activated oncogenes and their alterations are regarded as the optimal therapeutic target for drug screening and development, thereby overlooking the frequent loss of functional alterations in TSGs [18]. Recently, extensive attempts have been made to target somatic TSG alterations (e.g., loss‐of‐function mutations and CN deletion) through a synthetic lethality approach, resulting in the approval of several drugs by the US FDA, including PARP inhibitors for patients carrying *BRCA1*/*BRCA2* alterations [41]. However, the majority of commonly altered TSGs are not involved in synthetic lethality related molecular mechanisms as *BRCA1*/*BRCA2*. Therefore, a novel concept of “collateral lethality” was developed, synthetically lethally targeting the passenger deleted genes that are chromosomal proximal to the common deleted TSGs as points of selective vulnerability [18]. A series of studies have identified several TSG losses as candidates to confer vulnerability to their neighboring genes [23]. However, clinical applications for chemicals targeting these genes are not yet feasible. Therefore, we considered the combination of collateral lethality with another strategy (i.e., drug repurposing), which is a widely accepted and regarded as an attractive alternative to potentially accelerate precision oncology compared with novel drug development, as the drugs have already been approved for treatment of other diseases according to standard guidelines [42]. In this study, we discovered the chromosomal proximity between *NUDT15* and *RB1*, promoting us to design a combination of collateral lethality and drug repurposing therapeutic strategies for *RB1*‐deletion tumors using the US FDA‐approved substrate chemical of *NUDT15* (i.e., 6MP). Although previous studies have explored several collateral lethality targets, such as the RB1‐neighboring gene SUCLA2, no clinically validated drugs have been identified [23]. Additionally, we systematically screened candidate neighboring genes adjacent to other frequently deleted TSGs, such as TP53 and SMAD4, and found no suitable candidate genes comparable to NUDT15 that offer similarly robust rationales for collateral lethality paired with US FDA‐approved drugs. This highlights the uniqueness and clinical promise of targeting the *RB1*–*NUDT15* codeletion using 6MP.

*RB1* was the first cloned TSG, and its germline mutations with high penetrance account for half of retinoblastoma cases. Subsequently, common somatic alterations have been identified across different cancer types, particularly enriched in tumors with neuroendocrine features and linked to poor prognosis [8, 43, 44]. Moreover, the frequency of *RB1* alterations increases in tumors that acquired resistance to standard treatment regimens in a variety of cancer types, including CRPC [7]. As an example, *RB1* exhibited 100% loss in resistant *EGFR* mutant non‐small‐cell lung cancer that transformed to small‐cell lung cancer [45]. However, *RB1* was rarely considered as a direct therapeutic target for drug screening and strategy design due to the fact that the dominant truncating mutations and CN deletions of *RB1* in cancer render it untargetable. Alternatively, it is well established that *RB1* is involved in multiple tumor‐related pathways through its regulatory role on a number of proteins, such as the E2F family [46], suggesting that RB1 deficiency may be a relevant event for treatment stratification by targeting activated signaling. In fact, the possibility of using a synthetic lethality strategy to treat *RB1* deficient cancer has been evaluated. For example, the survival of *RB1* mutated small‐cell lung cancer cells is dependent on Aurora A and B [47, 48], thus providing a mechanical basis for candidate targets for *RB1* deficient tumors. Nevertheless, drug screening and evaluation will take a considerable amount of time before clinical applications can be made. In other studies, inhibition of CHK1 and PLK appears to have synthetic lethal effects in tumors with *RB1* deficiency, suggesting their inhibitors (e.g., volasertib) for selective vulnerability [49, 50]. In addition, a previous study shed light on the application of the collateral lethality strategy to treat *RB1*‐deleted tumors, revealing the possible passenger deleted neighbor gene *SUCLA2* as a potential target for *RB1* deletion [23]. However, as a result of screening with a library of natural compounds, the top candidate was thymoquinone, which is not a clinically proven chemical and therefore cannot be applied clinically quickly [23].

Interestingly, we found the close proximity of *NUDT15* and *RB1*, as well as their frequent somatic codeletions in various cancer types. This common genomic event confers a better candidate for collateral lethality because NUDT15 has been well characterized as a purine‐specific nucleotide diphosphatase that dephosphorylates the active metabolites of 6MP, an US FDA‐approved drug from the 1950s [24]. NUDT15 enzymatic deficiency induced by germline variants can increase the toxicity of 6MP to both leukemia and normal cells [24, 26]. As confirmed by our in vitro and in vivo experiments, it is possible to conduct repurposing 6MP to treat tumors with somatic codeletion of *RB1*–*NUDT15* that confer selective vulnerability. Moreover, systematic screening of drug sensitivity data from the DepMap database confirmed that, despite several drugs showing similar correlations with *RB1*–*NUDT15* deletions, 6MP remains the most clinically relevant candidate due to its established efficacy, safety profile, and existing clinical usage guidelines. Meanwhile, PRCA was selected as the primary disease model for functional validation of 6MP‐based collateral lethality owing to the high prevalence of RB1 CN deletions in this cancer type, as opposed to the predominance of RB1 mutations observed in other cancers such as breast or endometrial cancer. In addition, NUDT15 loss was not associated with poor prognosis in breast or esophageal cancer with current standard therapy, limiting the potential to prescribe 6MP to treat patients with these cancer types. However, this strategy is only applicable to *RB1* deletions but not loss‐of‐function mutations, limiting its applications to all patients with *RB1* deficiency. On the other hand, epigenetic silencing of *RB1* also frequently occurs in cancer [11], thus neighbor effects of expression correlation between *RB1* and *NUDT15* are induced by topologically associated domains [36, 37], thereby expanding the application of 6MP in patients with cosilencing of *RB1*/*NUDT15*. As indirect evidence, CDK4/6 inhibitors can suppress *RB1* expression [34, 35], and antagonize the anti‐tumor effects of thiopurine (i.e., 6MP, azathioprine, and thioguanosine), which may be induced by coactivated *NUDT15* expression [49]. Therefore, direct examination of NUDT15 and RB1 in tumors through a pathologic approach may guide the precise use of 6MP in RB1‐deficient patients.

Given somatic CN deletion‐induced *NUDT15* deficiency can sensitize cancer cells to 6MP, we hypothesized that tumors with somatic loss‐of‐function mutations of *NUDT15* could also be treated with 6MP. Similar to *NUDT15*, *TPMT* is another well‐established pharmacogenetic marker gene, germline variants of which also account for the intolerance of 6MP [27]. Therefore, it is possible to prescribe 6MP to tumors with *TPMT* deletions or loss‐of‐function mutations as well. However, the frequencies of either somatic CN deletions or mutations are low across different cancer types (Figure S15), probably because these alterations are neither causal to tumorigenesis nor passengers accompanied with common tumor‐related alterations.

Finally, due to the prevalence of germline variants that induce deficiencies of NUDT15 and TPMT, patient stratification is also necessary to avoid 6MP‐induced adverse drug reactions, particularly leucopenia [24, 27]. That is, genotyping the germline pharmacogenetic markers (i.e., *NUDT15* and *TMPT*) to estimate the metabolizer status of each patient according to the standard guideline [27]. Therefore, heterozygous carriers require a dosage adjustment, whereas homozygous carriers should not take 6MP for treatment because the somatic *NUDT15* deletion in tumor cells can acquire no additional 6MP‐related toxicity. In fact, an independent study employing different methodologies has also confirmed our hypothesis [51]. Furthermore, in line with our findings, their data indicate that 6‐MP treatment may also be effective in ovarian carcinomas harboring NUDT15–RB1 codeletions, extending the therapeutic potential beyond PRCA.

Nevertheless, our study has several limitations. First, although we experimentally validated our findings using PRCA cell lines and xenograft mouse models, other cancer types that also exhibit frequent *RB1*–*NUDT15* codeletions (particularly small‐cell lung cancer) were not experimentally assessed, limiting the generalizability of our results across diverse tumor entities. Second, the mechanistic impact of individual gene deletions (RB1 versus NUDT15) was not separately clarified through analyses using cell lines with *RB1*–*NUDT15* codeletion backgrounds complemented by exogenous expression of each gene, due to the absence of available PRCA cell lines harboring native *RB1*–*NUDT15* codeletions. Indeed, we also screened several common small‐cell lung cancer cell lines but found that they carried RB1 mutations rather than deletions. Finally, we did not systematically investigate the pharmacological mechanism underlying the observed sensitization effects. However, given that the biochemical mechanism by which NUDT15 modulates 6MP toxicity (through dephosphorylating DNA‐incorporated thioguanine nucleotides) is well established, we anticipate that future detailed pharmacological studies will further reinforce the rationale and application of 6MP‐based therapeutic strategies targeting *RB1*–*NUDT15* codeletion. Meanwhile, in vivo experiments were conducted in immunodeficient nude mice, limiting the assessment of potential immune‐mediated effects of 6MP, which warrants further investigation in syngeneic models with intact immune systems [52].

In conclusion, with a combination of collateral lethality and drug repurposing strategies, we exploited the fact that NUDT15 deficiency induces 6MP‐related adverse drug reactions, to treat tumors with codeletion of *RB1* and *NUDT15*. This study offers a novel approach and sheds light on the possibility of selectively targeting RB1‐deficient cells in the treatment of various types of cancer.

## 4 Materials and Methods

### 4.1 Bioinformatic Data Acquisition

CN, gene expression, and clinical data (e.g., survival follow‐up information) of patients from the TCGA project were downloaded from Xena (http://xena.ucsc.edu/) and cBioPortal (https://www.cbioportal.org/). Similar information in validation cohorts (MCTP, Broad/Cornell, GSE74685, MSKCC/DFCI, SU2C/PCF, Nguyen cohort, Chakraborty cohort, and Abida cohort, GSE54460, and GSE70769), gene expression in cell lines (GSE75620, GSE154190, GSE74620, GSE133568, and GSE177054), and single cell transcriptome matrices in PRCA/pan‐cancer (GSE176031 and GSE210347 [32]) was downloaded from cBioPortal (https://www.cbioportal.org/) or Gene Expression Omnibus (GEO), followed by information retrieval of *RB1*, *ITM2B*, *MED4*, and *NUDT15* for statistical analyses. Information on 6MP drug sensitivity and gene expression/ploidy score in 543 cancer cell lines was obtained from Depmap (https://depmap.org/portal/). Data from TCGA project were obtained on October 2, 2023.

### 4.2 Single Cell Transcriptome Analysis

Standard single cell data processing was performed according to our previous established pipelines based on well accepted methods [32, 53, 54, 55, 56, 57, 58], including filtering (based on cell count, gene count, mitochondrial ratio, and doublet), batch effect correction (based on FastMNN), clustering (based on Seurat), and visualization (based on UMAP). Single‐cell transcriptome based CN estimation, cell–cell communication, and cell trajectory analyses were performed by using inferCNV (https://github.com/broadinstitute/inferCNV), CellPhoneDB (https://github.com/Teichlab/cellphonedb), and Monocle2 (v2.12.0) respectively, details of which have been described in our pipeline [32, 53, 54].

### 4.3 Plasmid Construction for shRNA and Viral Particle Packaging

As the pipeline we described previously [59, 60], the scramble sequence or five shRNA sequences against *NUDT15* (Table S1) were cloned into pLKO.1 vector using *Eco*RI and *Age*I restriction enzymes (NEB, R3101, and R3552), and confirmed by Sanger sequencing. Lentiviral particles were prepared using the calcium chloride transfection method using 293T cells with the helper vectors. For cells in a 10 cm dish, 20 µg pLKO, 2 µg pCAGkGP1R, 2 µg pCAG4–RTR2, and 1 µg pCAG–VSV‐G were used for particle packaging.

### 4.4 Survival Analysis

Patients were divided into groups based on their gene‐level CN deletion status estimated by the GISTIC2 method (e.g., deep, shallow, and diploid) or the proper cutoff of gene expression for best separation in TCGA and validation cohorts. Survival analysis was performed through Kaplan‐Meier methods based on the R packages “survival” and “survminer” as we described previously [60, 61]. For the statistical significance of differences by groups, the log‐rank (Mantel–Cox) test was used. Data from TCGA project were obtained on October 2, 2023.

### 4.5 Statistical Analysis

For public data analysis, statistical analysis was performed in R v4.1.3. Pearson correlation, or Spearman rank correlation was used to measure the correlation between two continuous variables. For the statistical significance of differences by groups, the log‐rank (Mantel–Cox) test, Wilcoxon rank‐sum test and Student's *t*‐test were performed. And a *p* value less than 0.05 was considered statistically significant. For DEGs analysis, the false discovery rate was used to assess statistical significance.

For statistical analysis on experimental results (e.g., drug sensitivity), which were presented as the mean ± SEM, statistical analyses were performed using GraphPad Prism (version 9.0). When only two value sets were compared, the Student's *t*‐test was used for statistical analysis. One‐way ANOVA or two‐way ANOVA was conducted to test the mean difference between more than two groups. A *p* value less than 0.05 was considered statistically significant.

## Author Contributions

Conceptualization: Heng Xu. Data curation and formal analysis: Tao Zhou, Huancheng Fu, Zichen Zhao, Yiqi Deng, Hai‐Ning Chen, Wei‐Han Zhang, and Yang Shu. Funding acquisition: Heng Xu and Yan Zhang. Investigation: Huayun Yan, Dandan Yin, Yun Deng, Xiaoxi Lu, and Yunying Shi. Resources: Shuang Li, Yangjuan Bai, Pei Cai, and Lili Jiang. Supervision: Lanlan Wang, Qianqian Liu, Wei Zhang, Hong Wang, Yan Zhang, Bo Liu, and Heng Xu. Writing—original draft: Heng Xu and Tao Zhou. Writing—review and editing: all authors. All authors have read and approved the final manuscript.

## Ethics Statement

All animal experiments were conducted in accordance with protocols approved by the Institutional Animal Care and Use Committee of West China Hospital, Sichuan University (Approval No. 20230302070).

## Conflicts of Interest

The authors declare no conflicts of interest.

## Supporting information




**Supporting Information: Figure S1**:Frequencies of RB1 deletions in independent cohorts. **Supporting Information: Figure S2**: Impact of RB1 CN deletions on RB1 expression across different cancer types in TCGA. **Supporting Information: Figure S3**: Prognostic value of RB1 deletion and expression. **Supporting Information: Figure S4**: landscape of RB1 deletion at in prostate cancer single cell level. **Supporting Information: Figure S5**: Estimation of RB1‐NUDT15 deletion at in pan‐cancer single cell level. **Supporting Information: Figure S6**: Impact of NUDT15 CN deletions on NUDT15 expression across different cancer types in TCGA. **Supporting Information: Figure S7**: Prognostic value of NUDT15 deletion and expression. **Supporting Information: Figure S8**: Neighbor effect correlated gene expression in independent cohorts and control region. **Supporting Information: Figure S9**: IC50 correlation of chemicals in DepMap with the normalized copy number of NUDT15 or RB1. **Supporting Information: Figure S10**: Knockdown effects of NUDT15 shRNAs and safety dose estimation of 6MP in nude mice. **Supporting Information: Figure S11**: Knockdown effects of NUDT15 shRNAs and safety dose estimation of 6MP in nude mice. **Supporting Information: Figure S12**: Pathways affected by NUDT15 knockdown and 6MP treatment. **Supporting Information: Figure S13**: Knockdown effects of NUDT15 shRNAs on other 6MP metabolism. **Supporting Information: Figure S14**: Experimental validation of NUDT15 effect on mercaptopurine sensitivity. **Supporting Information: Figure S15**: Deep deletions and mutations frequencies of NUDT15 and TPMT across different TCGA cancer types. **Supporting Information: table S1**: Sequences for constructing five shRNAs against NUDT15. **Supporting Information: table S2**: qRT‐PCR primers for NUDT15 expression estimation.

## Data Availability

The data that support the findings of this study have been deposited into CNGB Sequence Archive (CNSA) [62] of China National GeneBank DataBase (CNGBdb) [63] with accession number CNP0007116.
