# Supplementary material for: Repurposing Mercaptopurine Through Collateral Lethality to Treat Cancers with Somatic RB1–NUDT15 Loss
Source: MedComm (2020). 2025 Sep 1;6(9):e70361. doi: 10.1002/mco2.70361 (PMC12402595; doi:10.1002/mco2.70361)
Supplement: Supplementary file 1 — Supporting Information: Figure S1:Frequencies of RB1 deletions in independent cohorts. Supporting Information: Figure S2: Impact of RB1 CN deletions on RB1 expression across different cancer types in TCGA. Supporting Information: Figure S3: Prognostic value of RB1 deletion and expression. Supporting Information: Figure S4: landscape of RB1 deletion at in prostate cancer single cell level. Supporting Information: Figure S5: Estimation of RB1‐NUDT15 deletion at in pan‐cancer single cell level. Supporting Information: Figure S6: Impact of NUDT15 CN deletions on NUDT15 expression across different cancer types in TCGA. Supporting Information: Figure S7: Prognostic value of NUDT15 deletion and expression. Supporting Information: Figure S8: Neighbor effect correlated gene expression in independent cohorts and control region. Supporting Information: Figure S9: IC50 correlation of chemicals in DepMap with the normalized copy number of NUDT15 or RB1. Supporting Information: Figure S10: Knockdown effects of NUDT15 shRNAs and safety dose estimation of 6MP in nude mice. Supporting Information: Figure S11: Knockdown effects of NUDT15 shRNAs and safety dose estimation of 6MP in nude mice. Supporting Information: Figure S12: Pathways affected by NUDT15 knockdown and 6MP treatment. Supporting Information: Figure S13: Knockdown effects of NUDT15 shRNAs on other 6MP metabolism. Supporting Information: Figure S14: Experimental validation of NUDT15 effect on mercaptopurine sensitivity. Supporting Information: Figure S15: Deep deletions and mutations frequencies of NUDT15 and TPMT across different TCGA cancer types. Supporting Information: table S1: Sequences for constructing five shRNAs against NUDT15. Supporting Information: table S2: qRT‐PCR primers for NUDT15 expression estimation. [file MCO2-6-e70361-s001.docx]

**Repurposing mercaptopurine through** **collateral lethality to treat cancers with somatic *RB1-NUDT15* loss**

Tao Zhou^1,2#^, Huayun Yan^2#^, Dandan Yin^2#^, Yun Deng^2^, Huancheng Fu^2^, Zichen Zhao^3^, Shuang Li^4^, Xiaoxi Lu^5^, Yiqi Deng^2,6^, Hai-Ning Chen^6,7^, Wei-Han Zhang^7,8^, Yunying Shi^9^, Yangjuan Bai^1^, Bei Cai^1^, Lanlan Wang^1^, Zhaoqian Liu^10^, Wei Zhang^10^, Lili Jiang^4^, Yang Shu^2,8^, Bo Liu^2^*, Yan Zhang^3^*, and Heng Xu^1,2,7,11^*

^1^ Department of Laboratory Medicine/Research Centre of Clinical Laboratory Medicine, West China Hospital, Sichuan University, Chengdu, Sichuan, China

^2^ State Key Laboratory of Biotherapy and Cancer Center, West China Hospital, Sichuan University, Chengdu, China

^3^ Lung Cancer Center/Lung Cancer Institute, Department of Medical Oncology, West China Hospital, Sichuan University, Chengdu, Sichuan, China

^4^ Department of Pathology, West China Hospital, Sichuan University, Chengdu, Sichuan, China

^5^ Department of Pediatric Hematology/Oncology, West China Second Hospital, Sichuan University, Chengdu, Sichuan, China

^6^ Colorectal Cancer Center, Department of General Surgery, West China Hospital, Sichuan University, Chengdu, Sichuan, China.

^7^ Institute of General Surgery, West China Hospital, Sichuan University, Chengdu, Sichuan, China.

^8^ Gastric Cancer Center, Department of General Surgery, West China Hospital, Sichuan University, Chengdu, Sichuan, China.

^9^ Department of Nephrology, West China Hospital, Sichuan University, Chengdu, Sichuan, China

^10^ Department of Clinical Pharmacology, Hunan Key Laboratory of Pharmacogenetics, Xiangya Hospital, Central South University, Changsha, Hunan, China

^11^ Tianfu Jincheng Laboratory, Chengdu, Sichuan, China.

**Key words:** RB1; NUDT15; copy number deletion; mercaptopurine; collateral lethality; drug repurposing

^#^ These authors contributed equally to this work

* To whom correspondence should be addressed

**Prof. Heng Xu**

Department of Laboratory Medicine/Research Center of Clinical Laboratory Medicine, State Key Laboratory of Biotherapy, West China Hospital, Sichuan University

No. 17, Section 3, South Renmin Road, Chengdu, Sichuan, China, 610041

Phone: +86-15928058711

Email: [xuheng81916@scu.edu.cn](mailto:xuheng81916@scu.edu.cn)

**Prof. Bo Liu**

State Key Laboratory of Biotherapy and Cancer Center, West China Hospital, Sichuan University

No. 17, Section 3, South Renmin Road, Chengdu, Sichuan, China, 610041

Phone: +86-15708469925

Email: [liubo2400@163.com](mailto:liubo2400@163.com)

**Prof. Yan Zhang**

Lung Cancer Center/Lung Cancer Institute, West China Hospital, Sichuan University

No. 37 Guoxue Alley, Chengdu, Sichuan, China, 610041

Phone: +86-18980606909

Yan Zhang: [zhang.yan@scu.edu.cn](mailto:zhang.yan@scu.edu.cn)

**Supplementary Methods**

**Immunohistochemistry (IHC)**

All Formalin-Fixed and Parrffin-Embedded samples were collected from 44 patients with CRPC at West China Hospital between Feb 2022 and May 2023, all specimens were obtained following the same standard procedure and validated by histopathological examination. RB1 (CAS: 13A10) and NUDT15 (CAS: A8368) antibodies for immunohistochemistry staining were procured from ZSGB-Bio and ABclonal Technology, respectively. Tissue samples were processed into uniform 4-5 µm sections after fixation, dehydration, and wax impregnation. Each sample was incubated with RB1 (ready-to-use) and NUDT15 (dilution ratio 1: 800) antibodies overnight at 4℃. After hematoxylin-eosin staining, they were compared and scored under a microscope by pathologist. IHC positivity was defined by the presence of clearly visible brown or dark-brown staining granules localized in the nucleus, cytoplasm, or cell membrane, depending on the expected localization pattern of each protein. Staining intensity and proportion of positively stained cells were scored, and samples were classified as RB1/NUDT15-loss or RB1/NUDT15-intact based on these staining scores in tumor cells. Positive staining in endothelial cells was considered as control for successful staining. This study was approved by the Ethics Committee of West China Hospital, Sichuan University (2018(280)). Written informed consent was obtained from all patients or their guardians, as appropriate.

**mIHC**

Multiplex immunofluorescence staining was carried out using the Opal™ 7-Color Manual IHC Kit (Akoya Biosciences, catalog No. NEL801001KT). Formalin-fixed paraffin-embedded tumor specimens, acquired from West China Hospital, Sichuan University, were sectioned into 5 µm slices. Following standard dewaxing in xylene and graded ethanol series, antigen retrieval was conducted by microwave heating in citrate buffer (pH 6.0) or EDTA buffer (pH 9.0) for 20 min. Sections were then incubated in blocking solution (Antibody Diluent/Block, Akoya Biosciences, catalog No. 72424205) for 10 min to minimize non-specific binding. Primary antibodies targeting Synaptophysin (ab32127, Abcam, 1:1600, Opal 620), RB1 (10048-2-IG, Proteintech, 1:400, Opal 570) and NUDT15 (A8368, ABclonal, 1:200, Opal 520) were subsequently applied. Samples were incubated with primary antibodies at room temperature for 1 h or at 4 °C overnight, followed by application of appropriate secondary antibodies and Opal fluorophore-conjugated tyramide signal amplification reagents (Opal 480, Opal 520, Opal 570, Opal 620, Opal 690; Akoya Biosciences; dilution 1:200) for 10 min. Between each staining cycle, microwave-based antigen retrieval was performed to remove residual antibody complexes and prepare tissues for subsequent staining. After completion of multiplex staining, sections underwent nuclear counterstaining with DAPI at room temperature for 5 min, mounted with anti-fade fluorescence mounting medium (Abcam, ab104135), and images were acquired using the Vectra Polaris Automated Quantitative Pathology Imaging System. Image analysis and quantification were conducted using QuPath software (version 0.4.3).

**Cell culture and construction of stable knockdown cell lines**

According to the standard methods we performed previously^1-3^, PC3 and HeLa cell lines were cultured in RPMI-1640 medium (Basal Median, L210KJ) supplemented with 10% FBS (EXCELL Bio, FSP500), 100U/mL penicillin, and 100μg/mL streptomycin (Basal Median, S110JV) at 37℃ with 5% CO_2_ in a humidified atmosphere. Stable knockdown was established by lentiviral transduction of shRNAs for 48 hours, followed by puromycin selection for an additional 48 hours. Cell lines were authenticated by karyotyping and cytogenetics and tested negative for mycoplasma contamination.

**Western blot**

Western blotting was performed to test the knockdown effect of NUDT15 in cell lines following standard methods as we described previously^1, 2^. Briefly, proteins were extracted from the incubated PC3 and HeLa cells using RIPA buffer, supplemented with protease and phosphatase inhibitors (Beyotime), and quantified through the BCA assay (Beyotime). The semiquantitative analysis was performed using β-actin and tubulin as reference proteins for loading control. Antibodies against NUDT15 (ABclonal, A8368, 1:1000), β-actin (Santa Cruz Biotechnology, sc-69879, 1:2000), CyclinD1 (HUABIO, ET1601-31, 1:2000), CDK2 (HUABIO, ET1602-6, 1:1000), CDK4 (Proteintech, 11026-1-AP,1:1000), p21 (Proteintech, 10355-1-AP,1:1000), p27 (Proteintech, 25614-1-AP,1:1000), CDK2 (Proteintech, 10122-1-AP,1:5000)，PARP1 (Proteintech, 13371-1-AP,1:1000) were used.

**RNA extraction and quantitative reverse PCR (qRT-PCR)**

Standard methods were performed as we described previously^3^. Total RNA was extracted from PC3 and HeLa cell lines using the RNA extraction Kit (74104; Qiagen), followed by reverse-transcription using the PrimeScript RT reagent Kit with gDNA Eraser (RR047A; Takara). The iTaq Universal SYBR Green mix (A25742; Life) was used to quantitate the mRNA levels of the indicated genes in triplicate on a CFX Connect Real-Time PCR Detection System (Bio-Rad). The primer sequences are listed in **Table S2**.

**Cell proliferation and clone formation assay**

A total of 2 × 10^4^ *NUDT15*-KD or control PC3/HeLa cells were seeded in 96 well plates for the cell proliferation assay. After the cells were adhered, 10 μl of CCK8 was added to each well, and the absorbance at 450 nm was measured after 3 hours of incubation and monitored daily thereafter until the end of day 7. Triplicates were performed at each timepoint, and experiments were repeated twice.

A total of 2 × 10^4^ *NUDT15*-KD or control cells in 90μl RPMI-1640 medium were seeded in 12 well plates. After the cells were adhered, 10 μl of 6MP (100ng/μl) was added to each well. After 6 days, all wells were fixed with paraformaldehyde for 30 min and stained with crystal violet. Finally, all wells were dissolved in 33% acetic acid solution and submitted to absorbance detection at 450 nm.

**Cellular drug sensitivity**

To determine thiopurine metabolism *in vitro*, we conducted a drug sensitivity assay following our previously reported method. A total of 5 × 10^6^ *NUDT15*-KD or control PC3/HeLa cells were treated with stepwise decreased concentrations of 6MP at 37 °C for 72 h. Similarly, 10 μL of CCK8 was added to each well at the end timepoint, followed by the absorbance measurement at 450 nm after 3 hours of incubation. Drug sensitivity estimation was assessed by calculating the cell survival rate and the IC_50_ of the drug based on the absorbance values. Triplicates were performed at each concentration point, and experiments were repeated three times.

**Flow cytometry**

Flow cytometry-based apoptosis assay was performed. The *NUDT15*-KD and control PC3 cells were pretreated with 6MP (50ng/μL) for 72 hours, and around 2 × 10^5^ cells were harvested and washed twice with PBS. Annexin-V/PI (YEASEN, 40305ES60) double staining was performed for 15 minutes and resuspended in 300ul PBS for detection by flow cytometry on Novo Cyte Flow Cytometer(2026R). Analyses were performed with FlowJo software (V10).

**Xenograft**

As per the standard methods we described previously, tumor xenograft models were constructed by subcutaneously inoculating nude mice with *NUDT15*-KD and control PC3/HeLa cells, with a cell inoculation number of 1 × 10^7^. The PC3 and HeLa cells were inoculated on male (n = 7 for each group) and female nude (n = 5 for each group) mice, respectively. Tumor volume and body weight of nude mice were measured every two days (formula for calculating tumor volume: volume = ab^2^/2, a represents the long diameter of the tumor, b represents the short diameter of the tumor). When the tumor volume reached about 200 mm^3^, daily 6MP (5mg/kg/day) was administered via intraperitoneal injection until the endpoints. All mice were sacrificed simultaneously when the maximal tumor volume reached 1000 mm^3^. Fresh peripheral blood was collected with anticoagulant (i.e., EDTA) immediately and submitted to a complete blood count test. Animal experiments of this study have been approved by Animal Ethical and Welfare Committee of West China Hospital (20230302070)

**RNA-seq**

NUDT15 knockdown and control cell lines derived from prostate cancer cell lines PC3 and 22RV1 were seeded in 6-well plates at densities of 6 × 10⁵ and 5 × 10⁵ cells per well, respectively. Once the cells adhered, they were treated with 6-mercaptopurine (6MP) at final concentrations of 30 ng/μL for PC3 and 0.5 ng/μL for 22RV1. After 48 hours of treatment, cells were harvested and total RNA was extracted using TRIzol reagent (Invitrogen, Carlsbad, CA, USA) according to the manufacturer's instructions. RNA purity and integrity were evaluated using a NanoDrop 2000 spectrophotometer (Thermo Fisher Scientific, Wilmington, DE, USA) and an Agilent 2100 Bioanalyzer (Agilent Technologies, Palo Alto, CA, USA), respectively. Subsequently, sequencing libraries were constructed following the standard protocol provided by Illumina, involving mRNA enrichment using oligo(dT)-coated magnetic beads, fragmentation of mRNA, reverse transcription to cDNA, second-strand synthesis, end-repair, A-tailing, adapter ligation, and PCR amplification. Libraries were validated and quantified using an Agilent Bioanalyzer and Qubit fluorometer (Thermo Fisher Scientific). Paired-end sequencing (2 × 150bp) was then performed on an Illumina NovaSeq X Plus platform (Illumina, San Diego, CA, USA). After sequencing, raw reads were quality-controlled and trimmed using FastQC (v0.12.1) and Trim Galore (v0.6.10) software, followed by mapping to the human genome (GRCh38/hg38) using HISAT2 (v2.2.1). with default alignment parameters. Aligned reads were quantified using featureCounts (v2.0.8) from the Subread package to generate gene-level count matrices based on the GENCODE annotation (release 38). Subsequent differential gene expression analyses were conducted using the limma (v3.58.1) package in R.

**Gene Set Enrichment Analysis**

To investigate the biological pathways affected by NUDT15 knockdown and 6MP treatment, we performed gene set enrichment analysis using the R package fgsea (v1.28.0). Differential expression data were generated from RNA-seq of PC3 and 22RV1 cells across different conditions. Genes were ranked based on log2 fold change and used as input for enrichment analysis. The KEGG pathway gene sets were obtained from the Molecular Signatures Database (MSigDB, c2.cp.kegg.v7.5.1.symbols.gmt). Enrichment scores and statistical significance (adjusted p-values) were calculated based on 10,000 permutations. Pathways with an adjusted p-value < 0.05 were considered significantly enriched.

**DNA methylation and enhancers analysis**

TCGA DNA methylation data (450K methylation microarray) for prostate adenocarcinoma (PRAD) were obtained from the UCSC Xena database (https://xena.ucsc.edu). To identify methylation probes potentially associated with RB1-NUDT15 co-regulatory enhancers, we first referred to the official annotation data of the methylation microarray provided by Illumina. Subsequently, we integrated ChIP-seq signals indicative of active promoter and enhancer histone modifications (H3K4me1, H3K4me3, and H3K27ac) from three prostate cancer cell lines (PC3, 22RV1, and VCaP), downloaded from CistromeDB (http://cistrome.org/db). Enhancer-associated probes were thus defined based on overlapping methylation probe coordinates with these histone modification peaks. Finally, we calculated Spearman’s correlation coefficients between the beta values of the identified enhancer-associated methylation probes and the corresponding expression levels of RB1 and NUDT15, aiming to validate their potential regulatory roles.

**Reference**

1. H Xu, X Zhao, D Bhojwani, et al. ARID5B Influences Antimetabolite Drug Sensitivity and Prognosis of Acute Lymphoblastic Leukemia. *Clinical Cancer Research*. 2020;26(1):256-264.

2. H-N Chen, Y Shu, F Liao, et al. Genomic evolution and diverse models of systemic metastases in colorectal cancer. *Gut*. 2022;71(2):322-332.

3. M Luo, Z Huang, X Yang, et al. PHLDB2 Mediates Cetuximab Resistance via Interacting With EGFR in Latent Metastasis of Colorectal Cancer. *Cellular and Molecular Gastroenterology and Hepatology*. 2022;13(4):1223-1242.


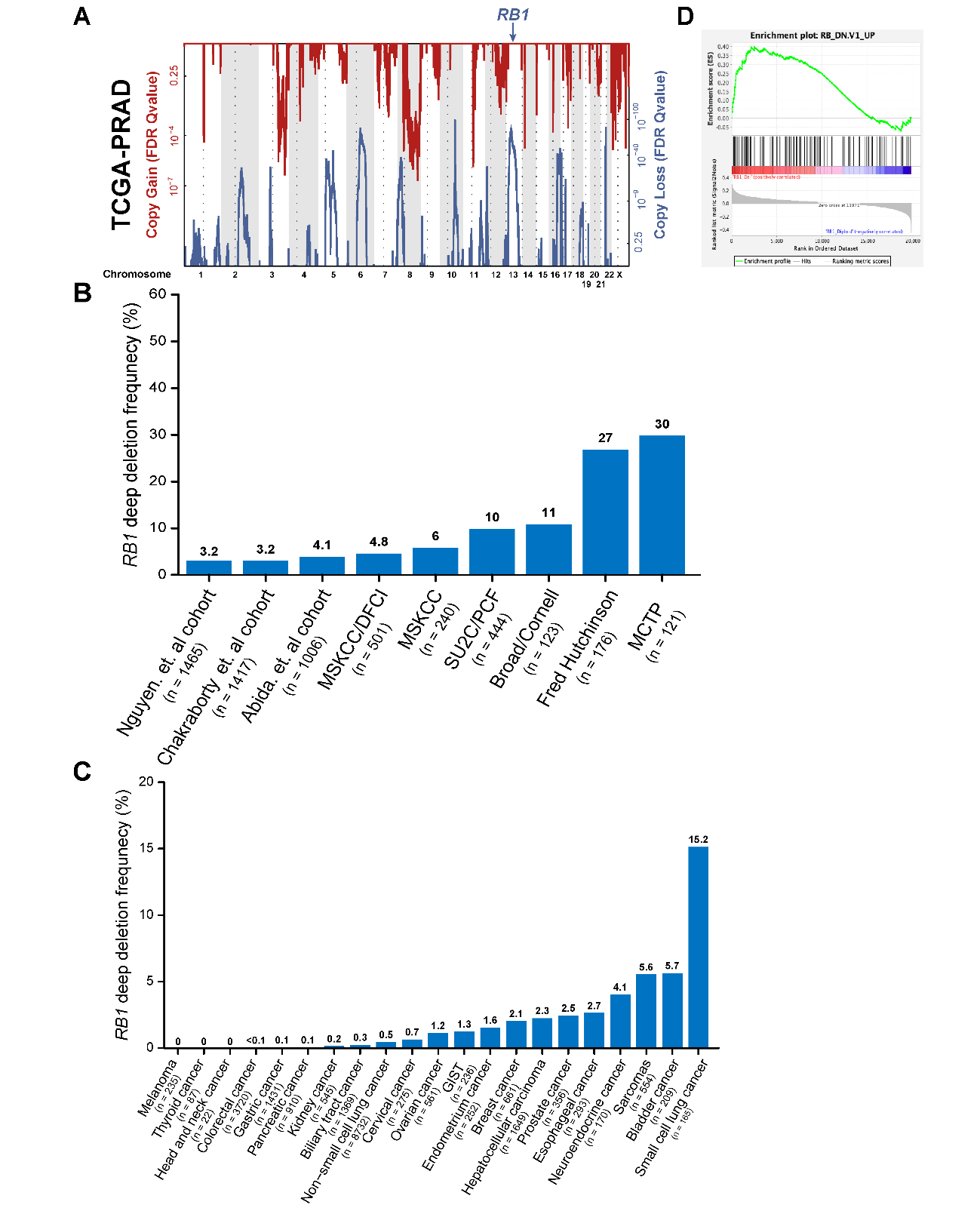


**Figure S1. Frequencies of *RB1* deletions in independent cohorts. (A)** Landscape of CN in prostate cancer; **(B)** Frequencies of *RB1* deletions in independent prostate cancer patient cohorts; **(C)** Frequencies of *RB1* deletions in various cancer types in Chinese patients; **(D)** Enrichment of RB pathway signaling in RB1 altered prostate cancer from TCGA according to GSEA.


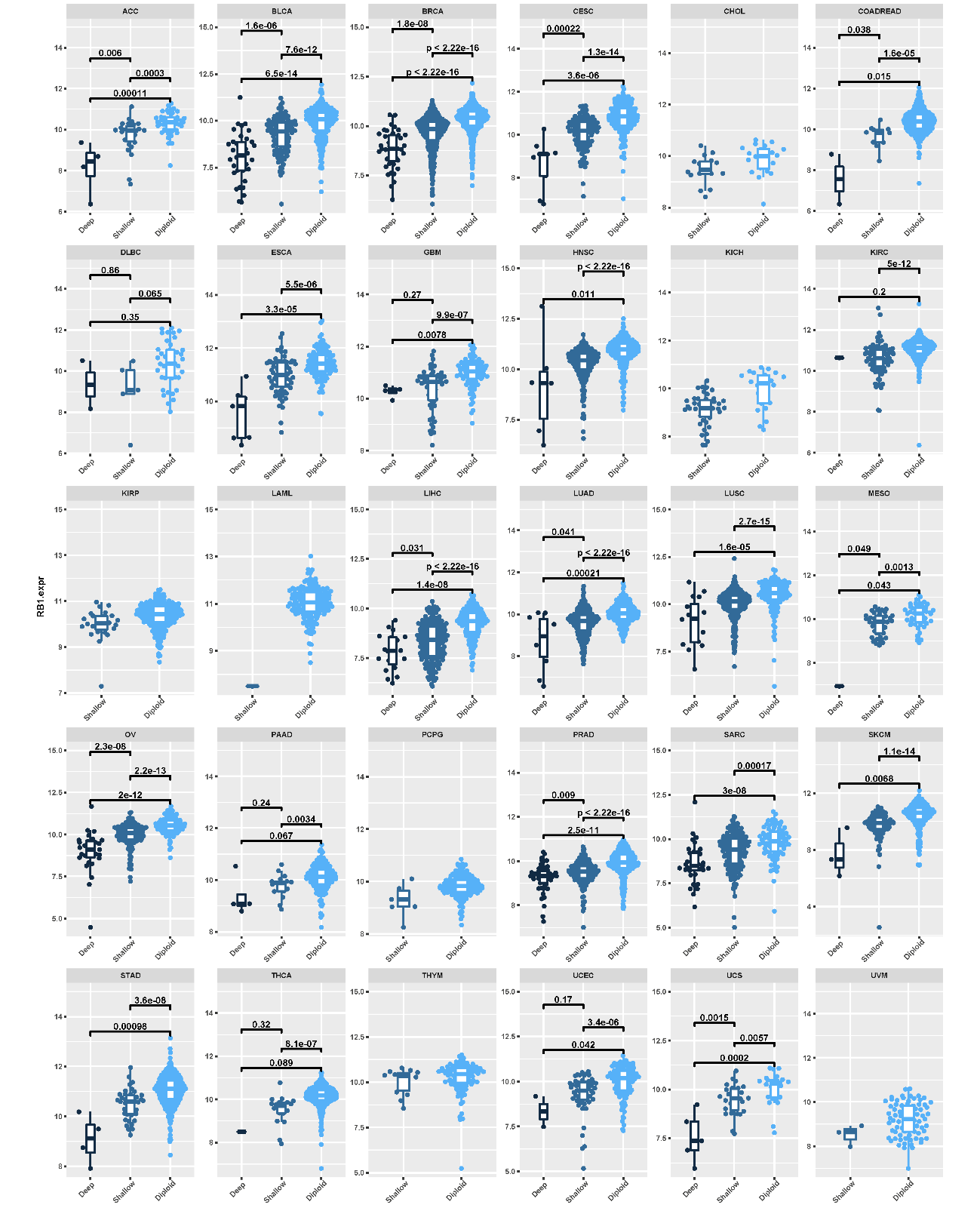


**Figure S2. Impact of *RB1* CN deletions on *RB1* expression across different cancer types in TCGA**


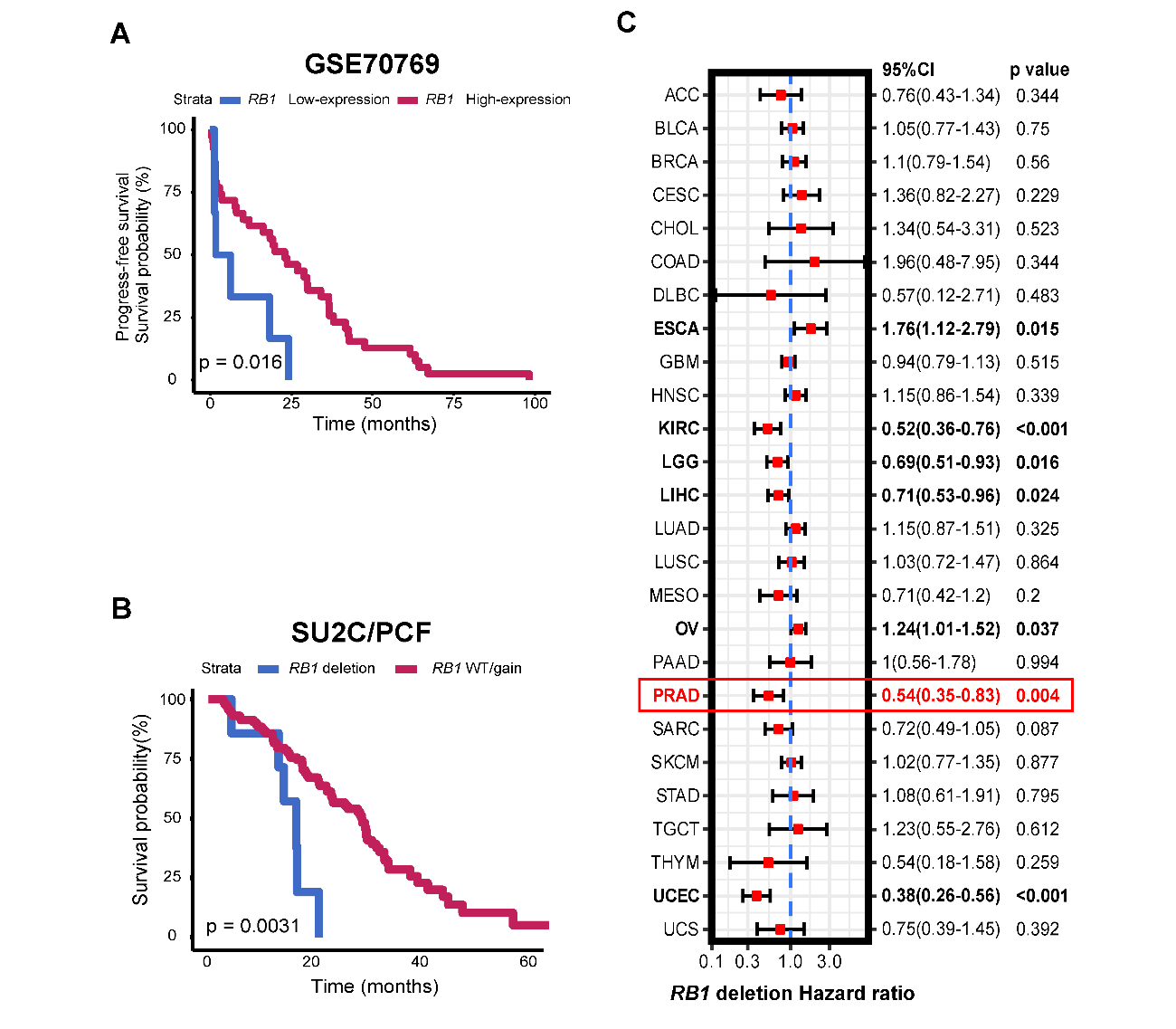


**Figure S3. Prognostic value of *RB1* deletion and expression. (A)** Prognostic association of *RB1* expression in cohort without CN deletion information (i.e., GSE70769); **(B)** Prognostic association of *RB1* CN deletion in independent prostate cancer cohort (i.e., SU2C/PCF); **(C)** Prognostic association of *RB1* deletion in different cancer types from TCGA. CN, copy number.


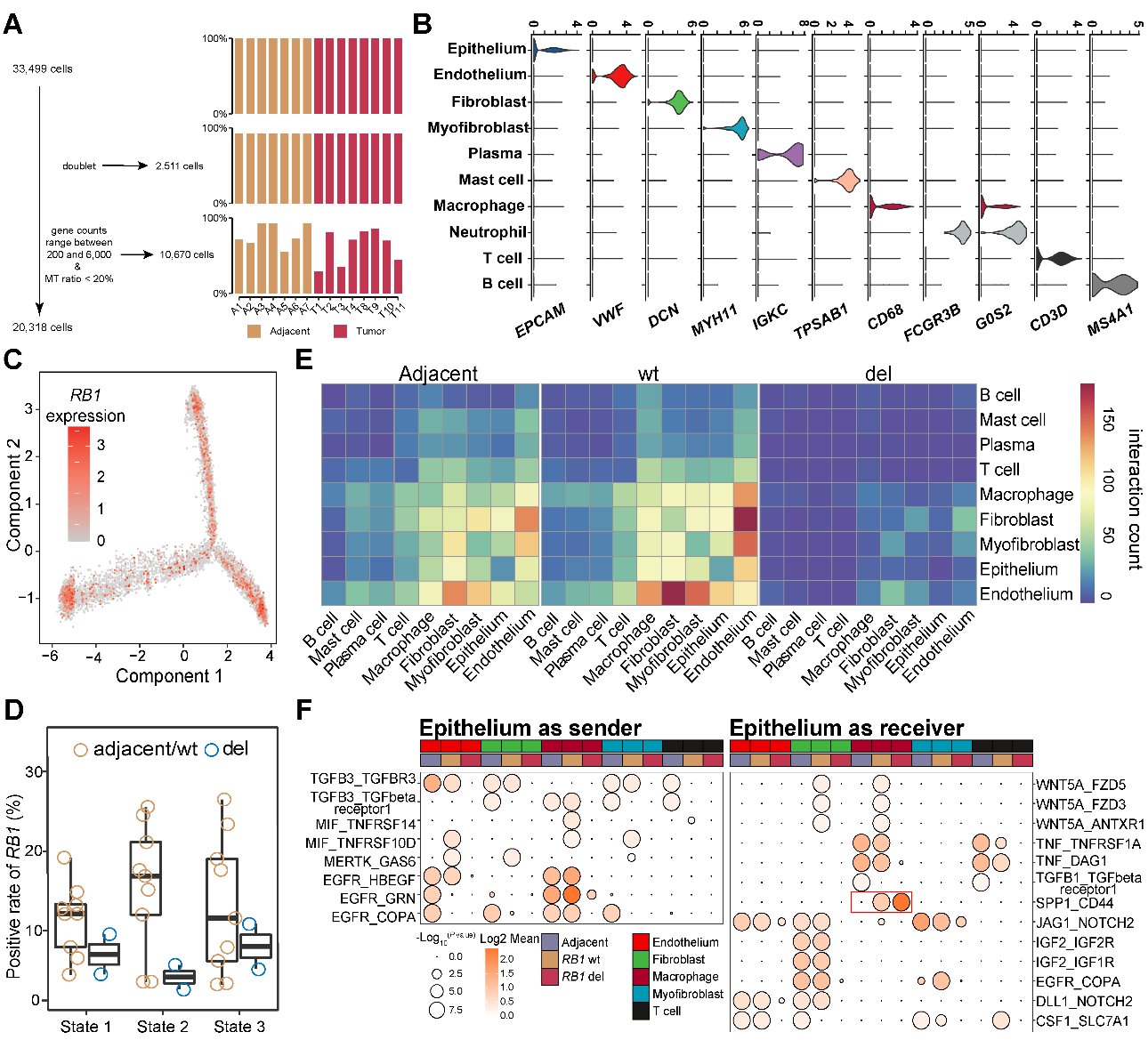


**Figure S4. landscape of *RB1* deletion at in prostate cancer single cell level. (A)** Quality control of single cell data of prostate cancer; **(B)** Expression of the canonical markers of each cluster illustrated by Violin plot; **(C)** *RB1* expression along the trajectory; **(D)** Comparison of *RB1*^+^ epithelial cells rate in each state separated by adjacent/wt *vs.* *RB1* deleted samples; **(E)** The predicted mutual interactions between epithelial cells with the main TME components in different sample origins; **(F)** Differential interactions of RB1 deleted samples between epithelial cells with other TME components. wt, CN-wildtype; del, deletion; chr13, chromosome 13.


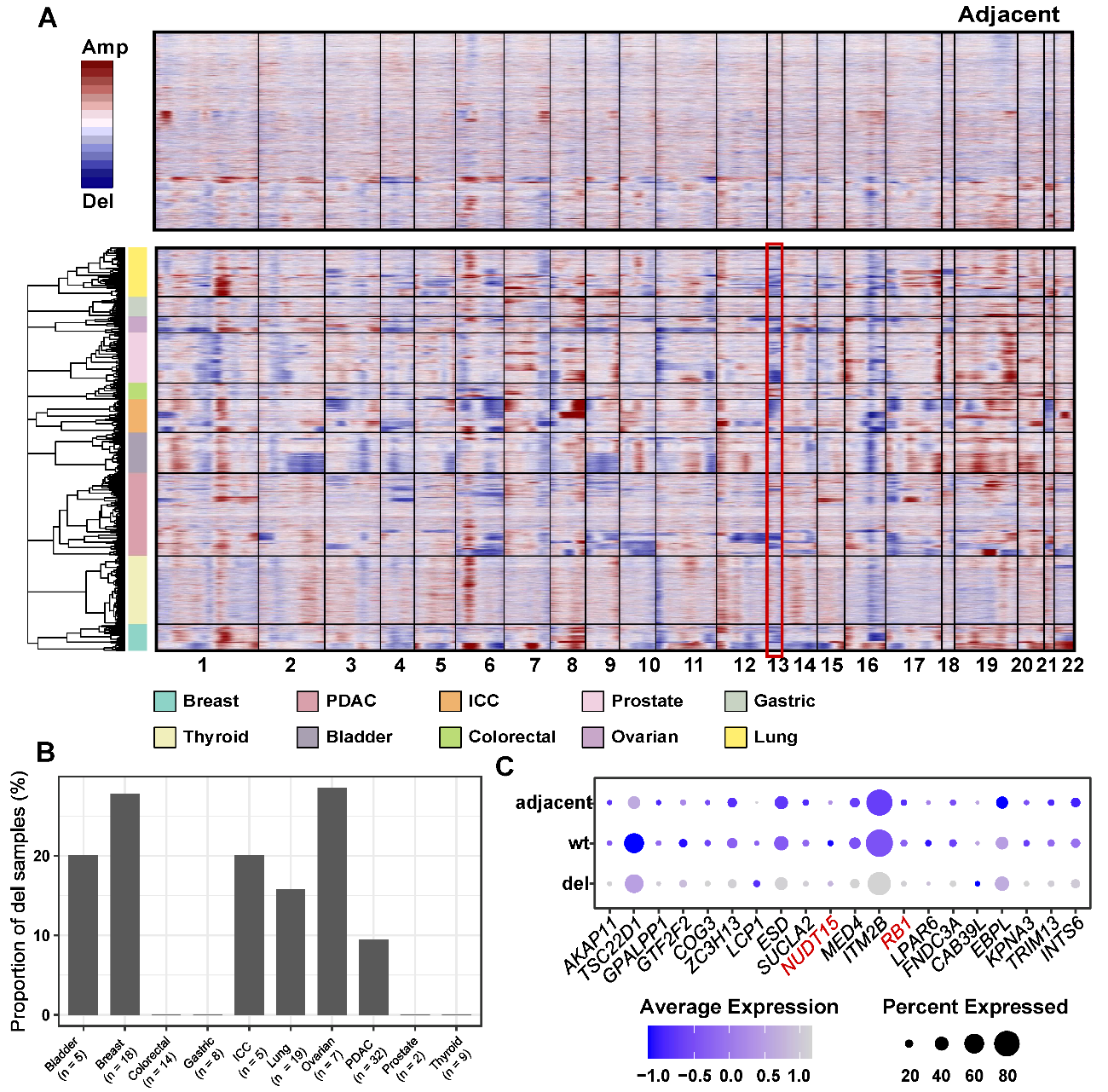


**Figure S5. Estimation of *RB1-NUDT15* deletion at in pan-cancer single cell level. (A)** CN estimated by InferCNV and the illustration of *RB1* deleted region in patients with pan-cancer single cell data; **(B)** proportion of estimated *RB1* deleted sample across cancer types with single cell data; **(C)** Illustration of *RB1*-deletion region in pan-cancer patients at single cell level.


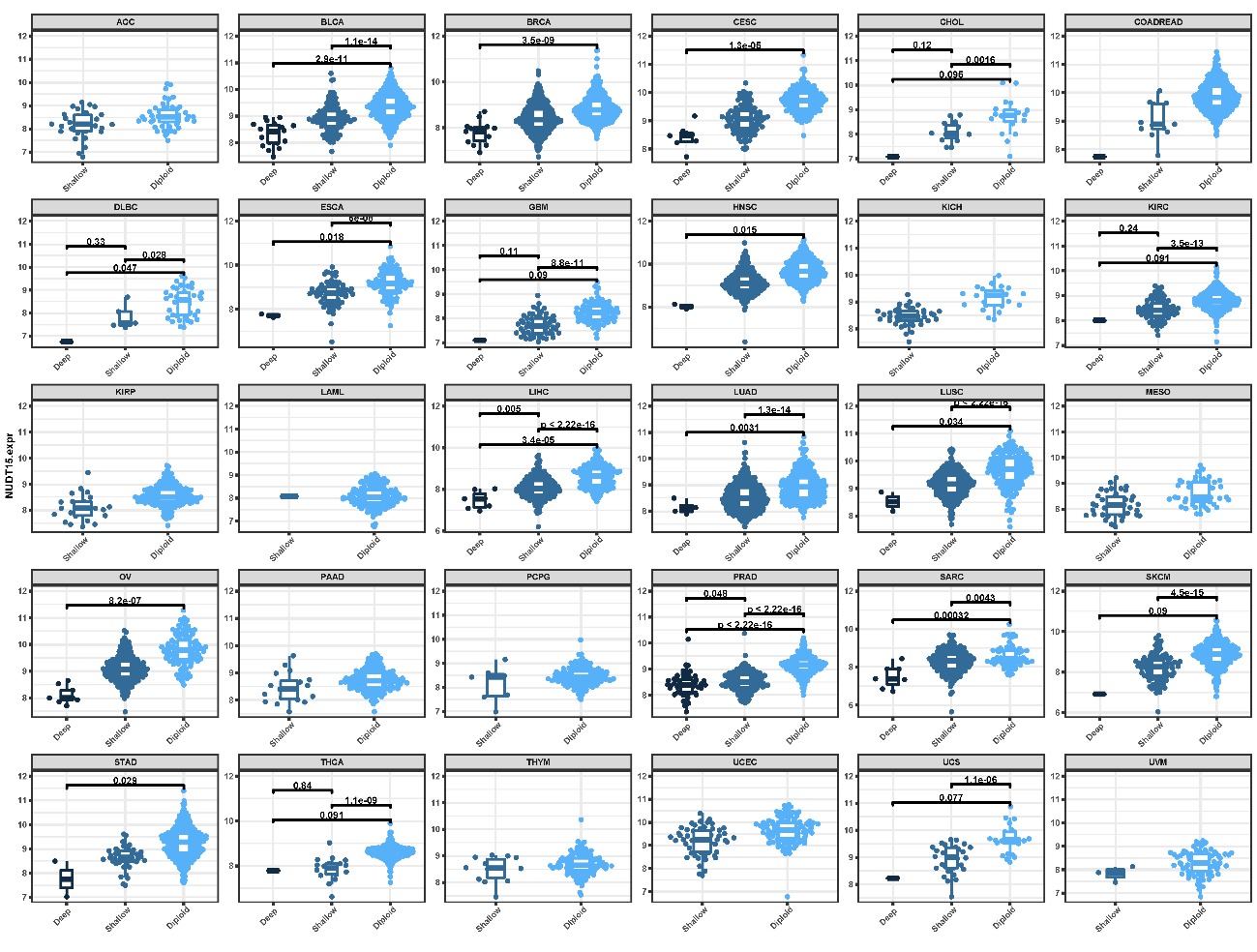


**Figure S6. Impact of *NUDT15* CN deletions on *NUDT15* expression across different cancer types in TCGA**


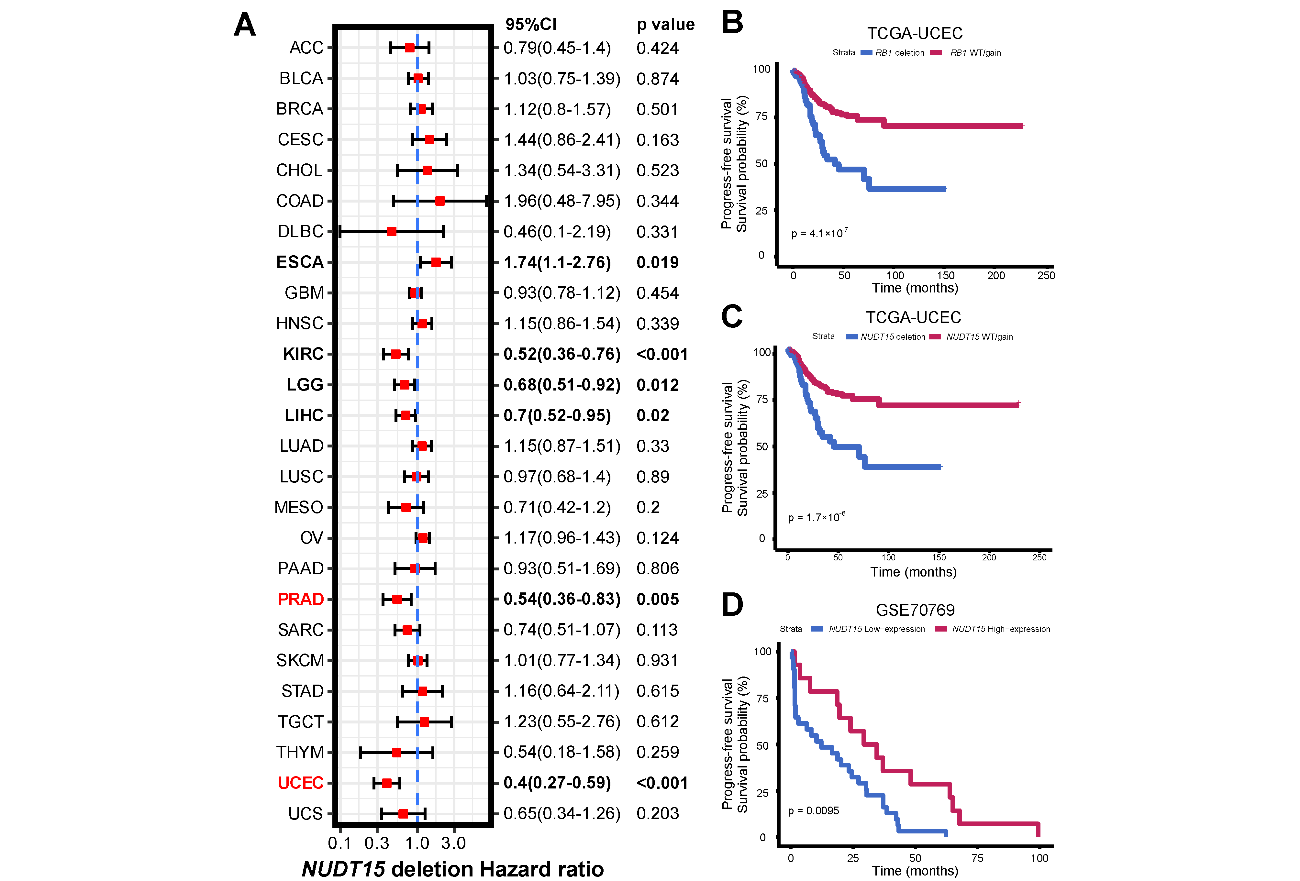


**Figure S7. Prognostic value of *NUDT15* deletion and expression. (A)** Prognostic association of *NUDT15* deletion in different cancer types from TCGA; **(B)** Prognostic association of *RB1* deletion in TCGA-UCEC; **(C)** Prognostic association of *NUDT15* deletion in TCGA-UCEC; **(D)** Prognostic association of *NUDT15* expression in independent prostate cancer cohort without CN deletion information (i.e., GSE70769)


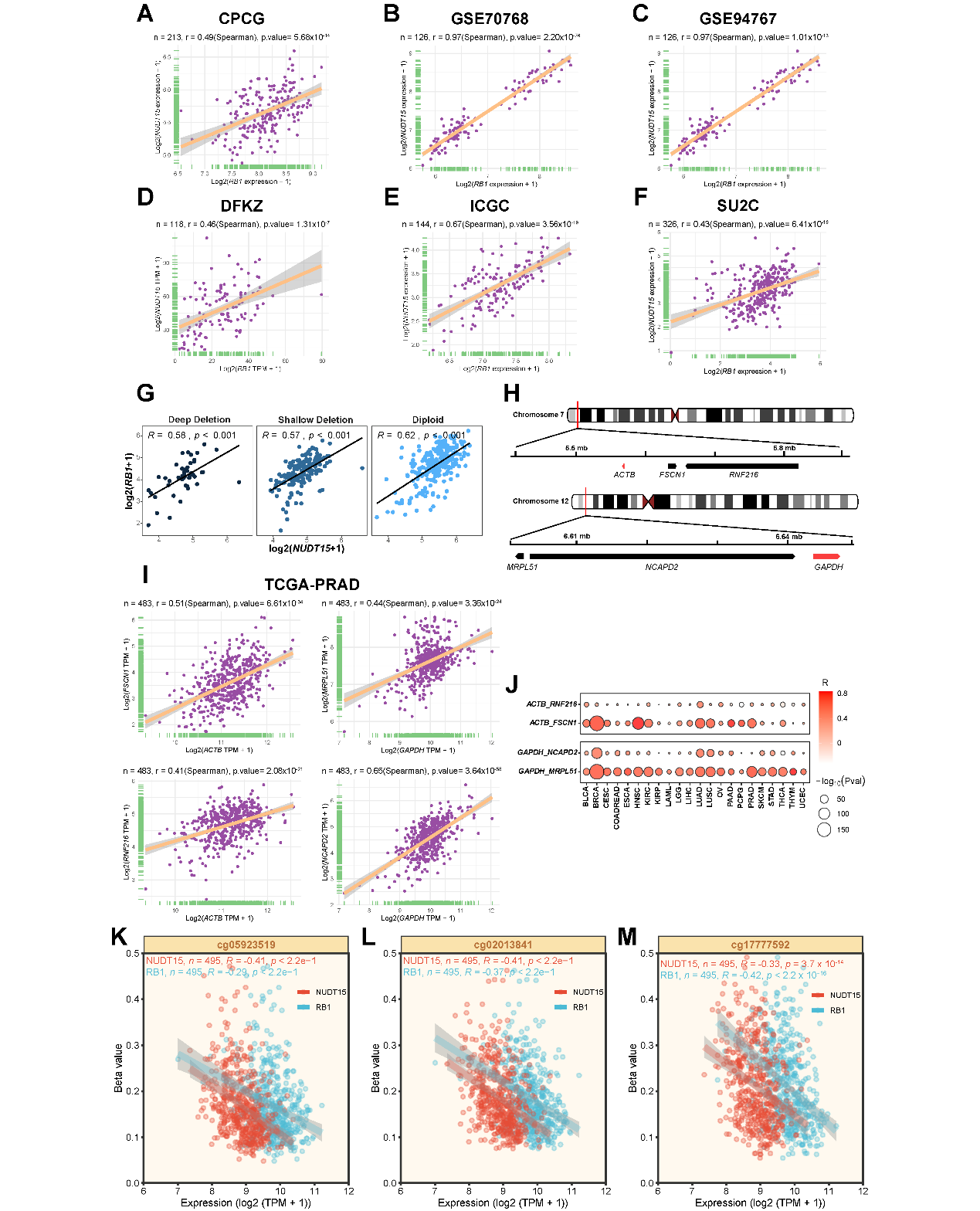


**Figure S8. Neighbor effect correlated gene expression in independent cohorts and control region. (A-F)** Expression correlation of *NUDT15* and *RB1* in independent prostate cancer cohorts; **(G)** Expression correlation of *NUDT15* and *RB1* in TCAG-PRCA patients with different *RB1* deleted status; **(H)** Illustration of neighbor genes of *ACTB* and *GAPDH***; (I)** Expression correlation of *ACTB*/*GAPDH* with their neighbor genes in TCGA-PRCA; **(J)** Expression correlation of *ACTB*/*GAPDH* with their neighbor genes across cancer types in TCGA. **(K-M)** Correlation analysis between DNA methylation levels at the identified enhancer regions and the expression of RB1 and NUDT15.


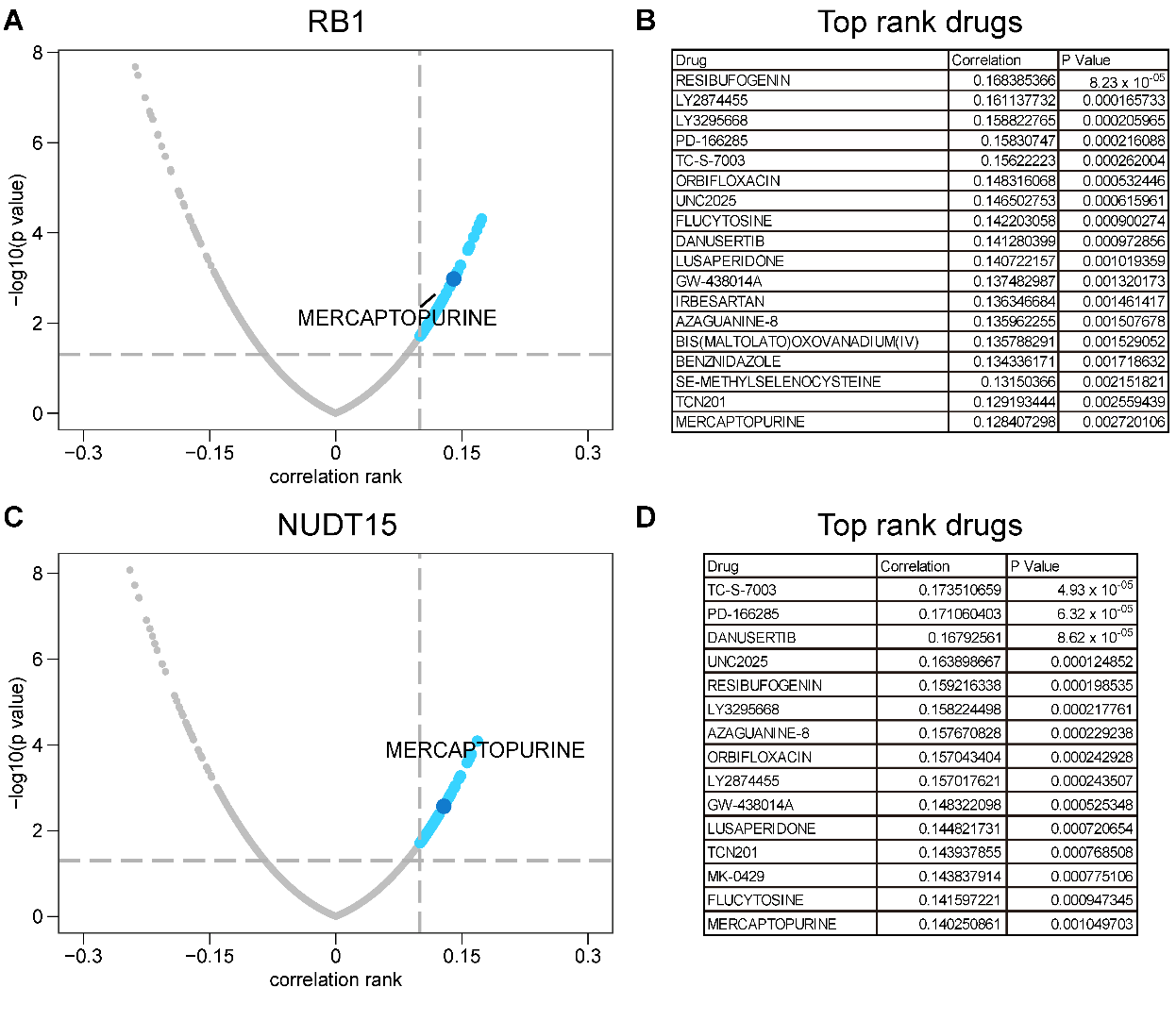


**Figure S9. IC50 correlation of chemicals in DepMap with the normalized copy number of NUDT15 or RB1. (A-B)** IC50 correlation rank of chemicals in DepMap with the normalized copy number of RB1; **(C-D)** IC50 correlation rank of chemicals in DepMap with the normalized copy number of NUDT15;


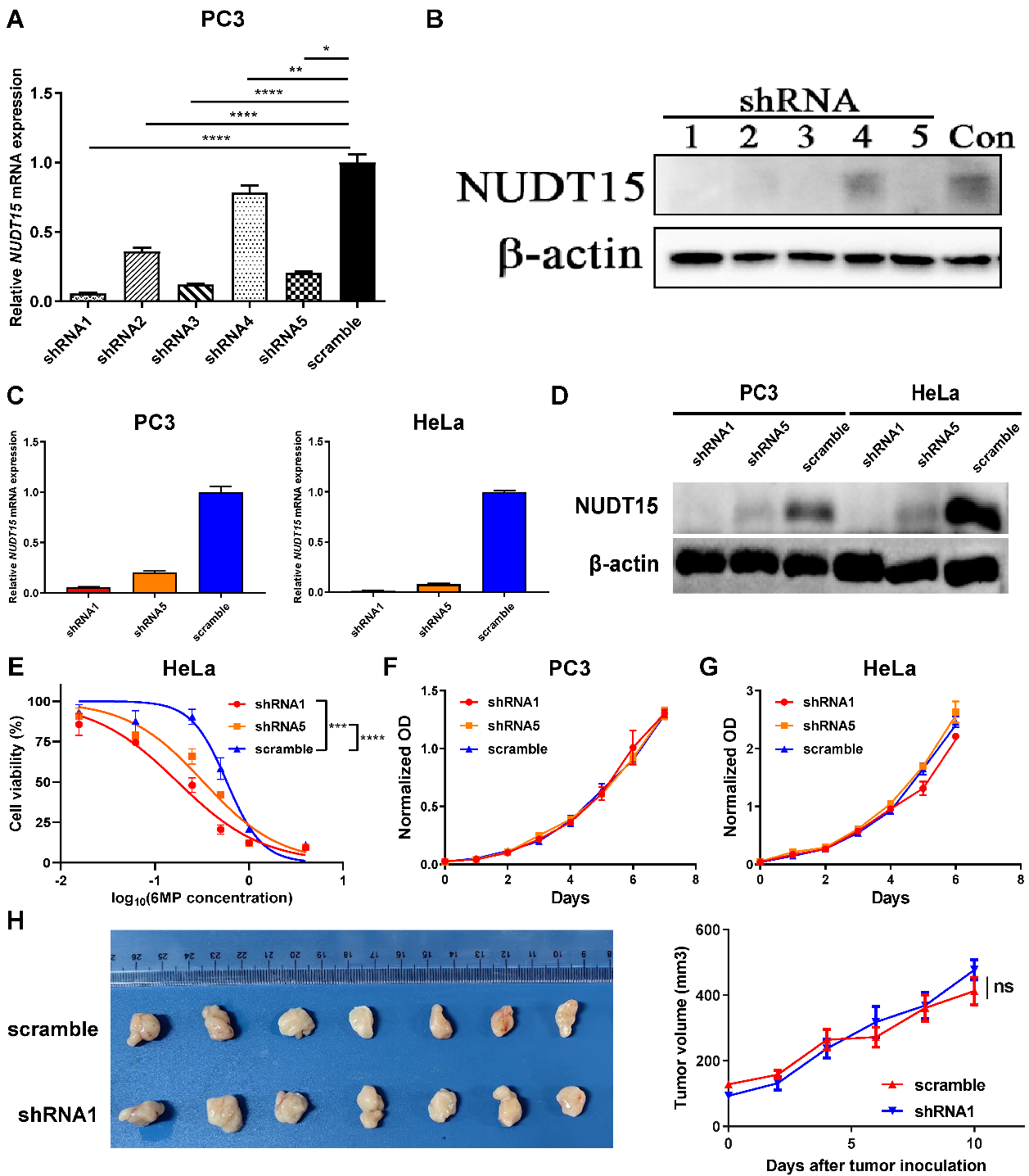


**Figure S10. Knockdown effects of *NUDT15* shRNAs and safety dose estimation of 6MP in nude mice. (A)** Estimation of *NUDT15* knockdown efficacy by using five shRNAs through q-PCR; **(B)** Estimation of *NUDT15* knockdown efficacy by using five shRNAs through western blot; **(C)** Estimation of *NUDT15* knockdown efficacy of shRNA1 and shRNA5 through q-PCR; **(D)** Estimation of *NUDT15* knockdown efficacy of shRNA1 and shRNA5 through western blot; **(E)** Mercaptopurine sensitivity evaluation on NUDT15 KD/control in HeLa cell lines, three replicates were performed for each concentration; **(F-G)** Cell proliferation evaluation on NUDT15 KD/control in PC3 and HeLa cell lines, three replicates were performed for each time points; **(H)** Illustration of the final tumor and determination of tumor volume with NUDT15 KD/control PC3 cells (n = 5); *, *p* < 0.05; **, *p* < 0.01; ***, *p* < 0.001; ****, *p* < 0.0001;


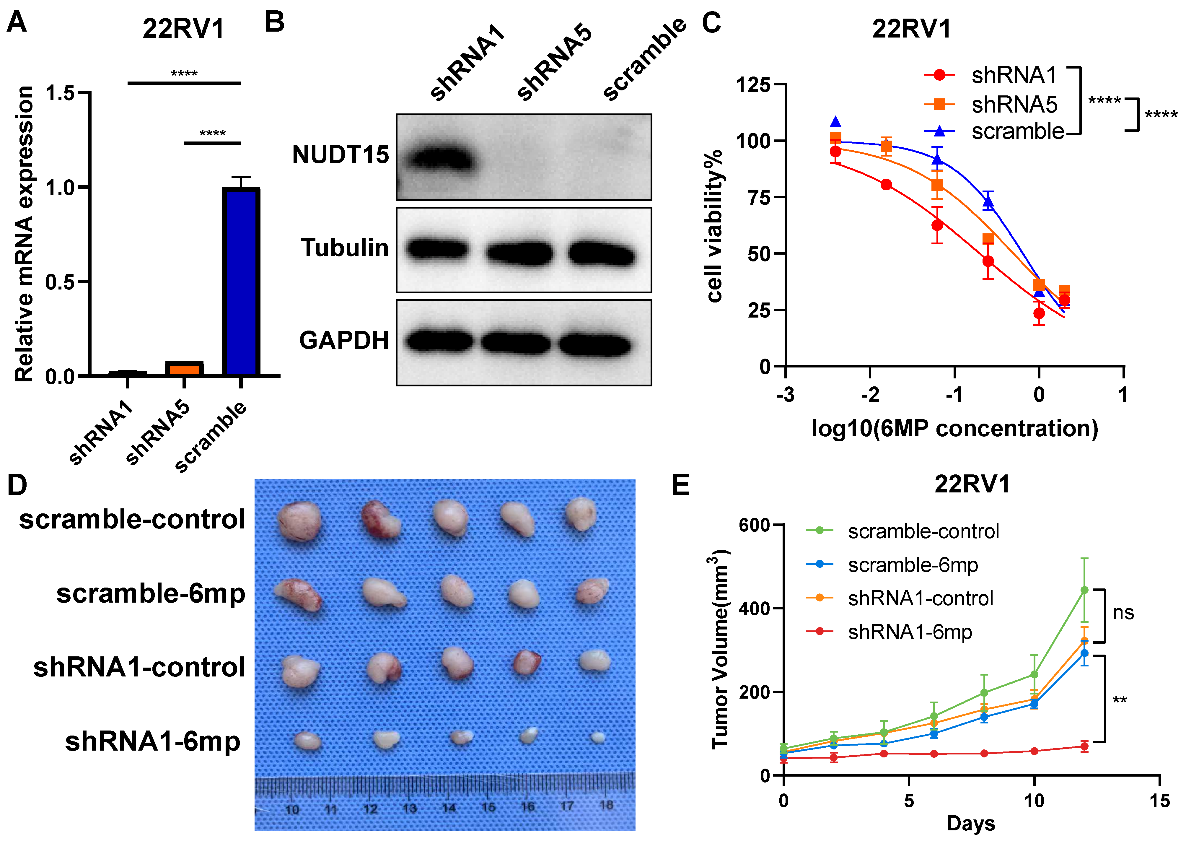


**Figure S11. Knockdown effects of *NUDT15* shRNAs and safety dose estimation of 6MP in nude mice.** **(A-B)** Estimation of *NUDT15* knockdown efficacy of shRNA1 and shRNA5 through q-PCR and western blot; **(C)** Mercaptopurine sensitivity evaluation on NUDT15 KD/control in 22RV1 cell lines, three replicates were performed for each concentration; **(D-E)** Illustration of the final tumor and determination of tumor volume with NUDT15 KD/control 22RV1 cells (n = 5). *, *p* < 0.05; **, *p* < 0.01; ***, *p* < 0.001; ****, *p* < 0.0001;


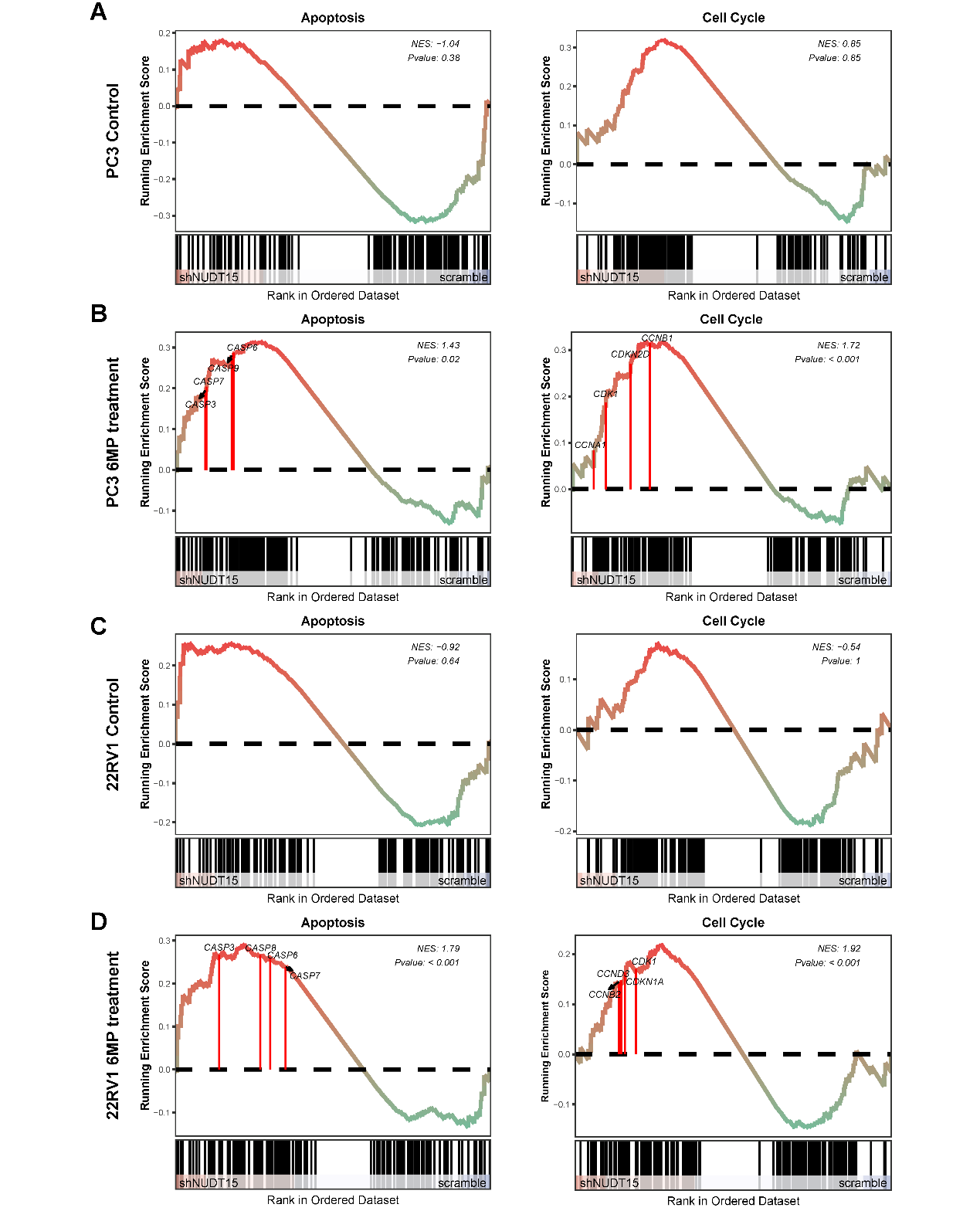


**Figure S12. Pathways affected by NUDT15 knockdown and 6MP treatment. (A-B)** Significantly enriched pathways of RNA-seq data from PC3 cell line under different conditions; **(C-D)** Significantly enriched pathways of RNA-seq data from 22RV1 cell line under different conditions. Normalized enrichment scores (NES) and p-values are shown for representative pathways.


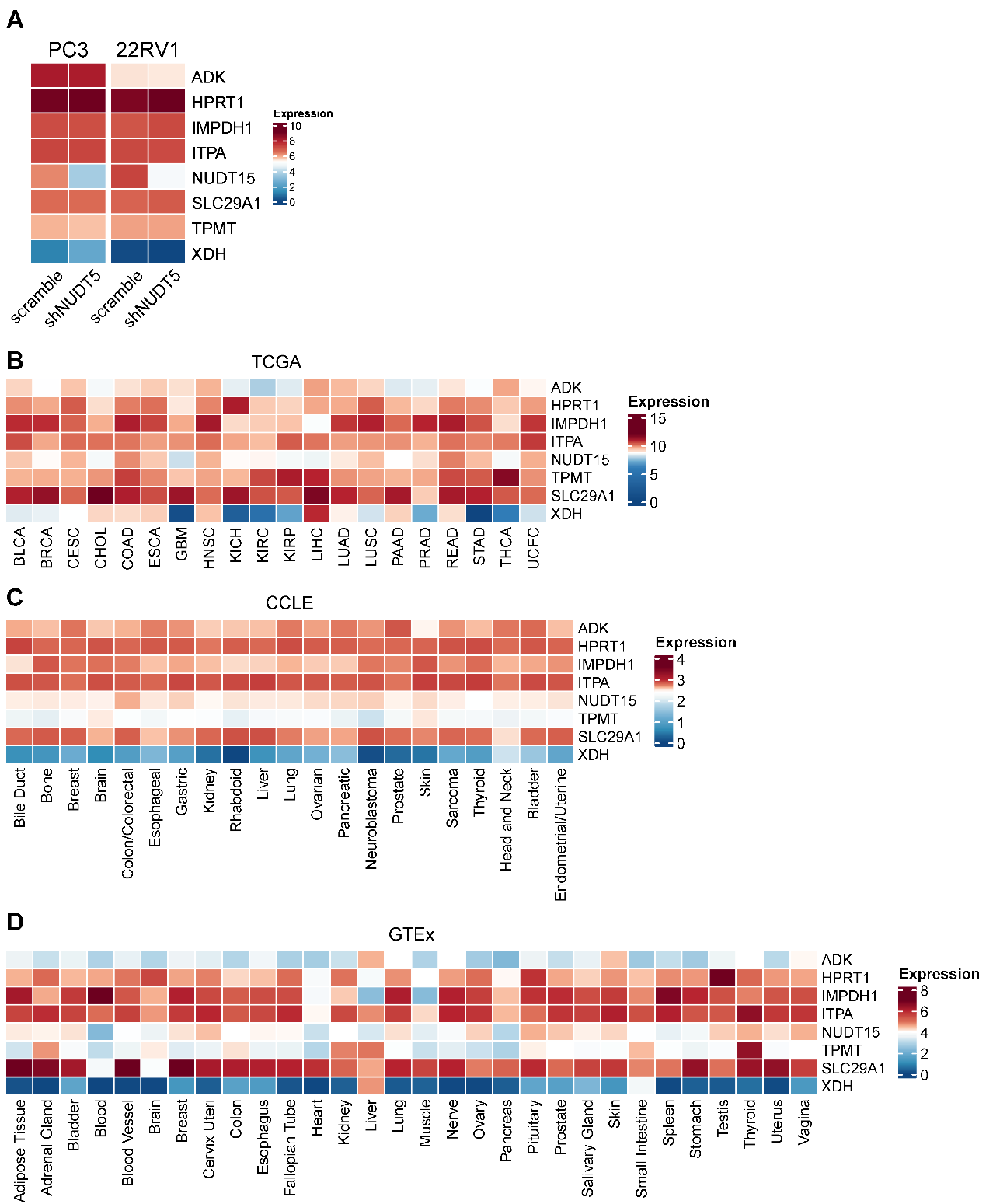


**Figure S13. Knockdown effects of *NUDT15* shRNAs on other 6MP metabolism** enzymes**. (A)** Estimation of 6MP metabolism enzymes after *NUDT15* knockdown through RNA-seq; **(D-E)** Expression of 6MP metabolism enzymes in different cancer types based on TCGA, CCLE, GTEx database;


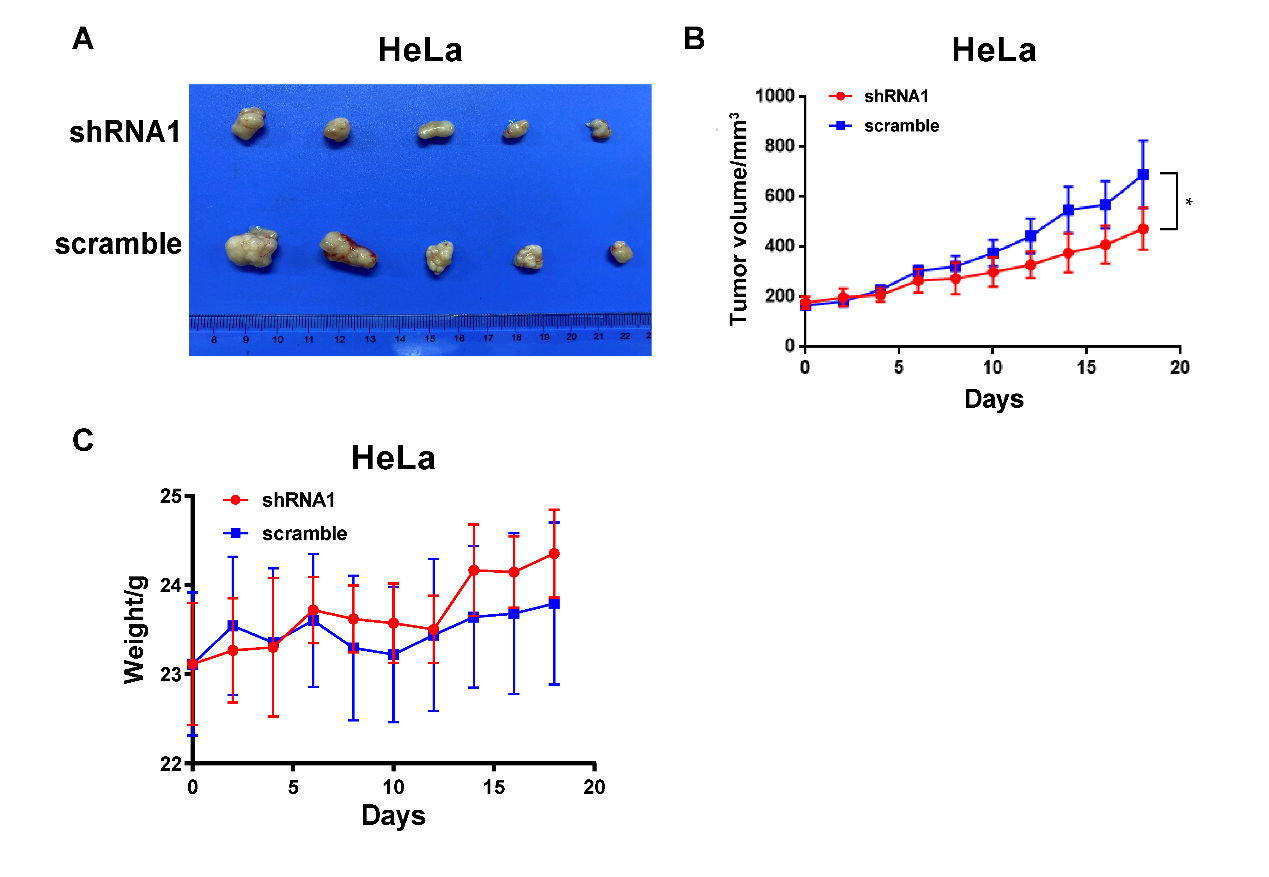


**Figure S14. Experimental validation of NUDT15 effect on mercaptopurine sensitivity.** **(A-B)** Illustration of the final tumor and determination of tumor volume with NUDT15 KD/control HeLa cells (n = 5); **(C)** Evaluation of body weight at the end points before sacrificing the nude mice. 6MP, mercaptopurine.


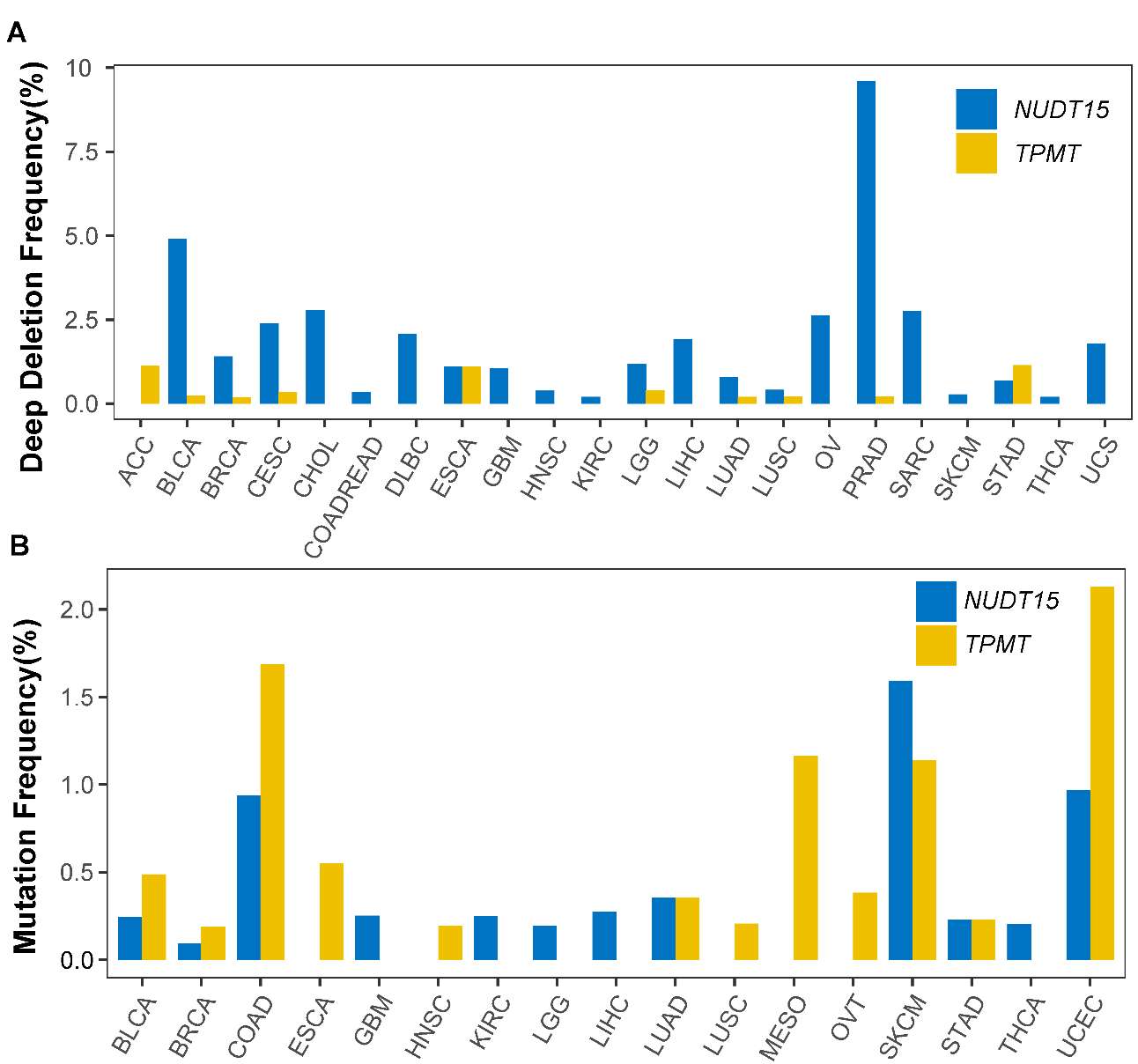


**Figure S15. Deep deletions and mutations frequencies of *NUDT15* and *TPMT* across different TCGA cancer types. (A)** Frequencies of *NUDT15* and *TPMT* deletion; **(B)** Frequencies of NUDT15 and TPMT non-silent mutations.

**Table S1. Sequences for constructing five shRNAs against *NUDT15***

| **shRNA primer** | **shRNA sequence** |
| --- | --- |
| NUDT15-shRNA-F1 | AATTCAAAAAGAAGCAGCTCTTCACCTGAAACTCGAGTTTCAGGTGAAGAGCTGCTTC |
| NUDT15-shRNA-R1 | CAAAAAGAAGCAGCTCTTCACCTGAAACTCGAGTTTCAGGTGAAGAGCTGCTTCCCGG |
| NUDT15-shRNA-F2 | AATTCAAAAAGACTCATGATTCAGAACCAAACTCGAGTTTGGTTCTGAATCATGAGTC |
| NUDT15-shRNA-R2 | CCGGGACTCATGATTCAGAACCAAACTCGAGTTTGGTTCTGAATCATGAGTCTTTTTG |
| NUDT15-shRNA-F3 | AATTCAAAAACAAGGCTATGATCCATTTAAACTCGAGTTTAAATGGATCATAGCCTTG |
| NUDT15-shRNA-R3 | CCGGCAAGGCTATGATCCATTTAAACTCGAGTTTAAATGGATCATAGCCTTGTTTTTG |
| NUDT15-shRNA-F4 | AATTCAAAAACTGGTGGGATACAAAGGAAATCTCGAGATTTCCTTTGTATCCCACCAG |
| NUDT15-shRNA-R4 | CCGGCTGGTGGGATACAAAGGAAATCTCGAGATTTCCTTTGTATCCCACCAGTTTTTG |
| NUDT15-shRNA-F5 | AATTCAAAAACCTGGGAAGAATGTGCTCAAACTCGAGTTTGAGCACATTCTTCCCAGG |
| NUDT15-shRNA-R5 | CCGGCCTGGGAAGAATGTGCTCAAACTCGAGTTTGAGCACATTCTTCCCAGGTTTTTG |

**Table S2. qRT-PCR primers for NUDT15 expression estimation.**

| Primer name | Primer sequence |
| --- | --- |
| NUDT15-qPCR-F | GAAAGGAGAAGTGGATGTGAC |
| NUDT15-qPCR-R | GGAACCCACTCCCAACTTTC |
| TUBULIN-qPCR -F | TGGACTCTGTTCGCTCAGGT |
| TUBULIN-qPCR -R | TGCCTCCTTCCGTACCACAT |
